# The Relationship Between Schizotypy and Latent Inhibition and Conditioned Inhibition

**DOI:** 10.1093/schizbullopen/sgag031

**Published:** 2026-07-31

**Authors:** Liam Myles, Lucy Cheke, Jane Garrison

**Affiliations:** Department of Psychology, University of Cambridge, Cambridge CB2 3EB, United Kingdom; Department of Psychology, University of Cambridge, Cambridge CB2 3EB, United Kingdom; Department of Psychology, University of Cambridge, Cambridge CB2 3EB, United Kingdom

**Keywords:** schizophrenia, perceptual learning, salience hypothesis, latent inhibition, conditioned inhibition

## Abstract

The *Salience Hypothesis* suggests that people high in symptoms of schizophrenia aberrantly assign salience. Studies of this theory are typically limited by confounds of learned irrelevance, conditioned inhibition (CI), negative priming, and novel pop-out effects. This study circumvented these confounds by using a perceptual learning (PL) paradigm to examine the effect of schizophrenia symptoms and hallucination proneness (HP) on latent inhibition (LI) and CI. Two hundred sixty participants completed the Oxford-Liverpool Inventory of Feelings and Experiences and the Multi-Modality Unusual Sensory Experiences Questionnaire, before completing a PL task. In this task, participants received pre-exposure to 2 highly similar checkerboards, either in separate blocks or an intermixed fashion. Participants were serially presented with pairs of either pre-exposed or novel checkerboards and determined whether they were different. Participants discriminated between similar checkerboards more quickly after pre-exposure, with similar reaction times (RTs) after intermixed and blocked pre-exposure. Higher unusual beliefs and lower HP predicted poorer accuracy following blocked pre-exposure. Greater unusual beliefs and lower cognitive disorganization predicted slower RTs after mixed pre-exposure. These results suggest that greater unusual beliefs and lower HP are associated with attenuated LI. Furthermore, they indicate that higher unusual beliefs and lower cognitive disorganization are associated with attenuated CI.

## Introduction

Schizophrenia is a serious mental health condition that affects 0.29% of people^1–3^ and is characterized by hallucinations and “delusional” beliefs.^4^ “Schizotypy” refers to a continuum of symptoms associated with schizophrenia, including “unusual experiences,” “introvertive anhedonia,” “cognitive disorganisation,” and “impulsive non-conformity.”^5–8^

The *Salience Hypothesis* is an influential theory of schizophrenia. It argues that people with schizophrenia aberrantly assign salience, culminating in familiar stimuli of otherwise low significance maintaining a sense of prominence.^9–13^ This theory suggests that hallucinations stem from aberrantly salient internal, self-generated stimuli (i.e., thoughts, mental images and/or memories), and that unusual beliefs arise as one generates explanations for hallucinations.

Evidence for the Salience Hypothesis comes from research on *latent inhibition* (LI). “Latent inhibition” refers to the phenomenon that exposure to a conditioned stimulus (CS) impedes subsequent learning about the relationship between that CS and an unconditioned stimulus (US).^14^^,^^15^ Some theorists suggest that the CS’s salience diminishes during pre-exposure, attenuating its associability (i.e., the ease with which it is conditioned) during the conditioning phase.^13–21^ As the salience hypothesis asserts that stimuli maintain greater salience in people with schizophrenia, researchers have examined whether LI is attenuated in people with schizophrenia.

In our review of this field, we found that most studies suggested that schizophrenia and elevated schizotypy are associated with diminished LI.^22^ Indeed, several studies suggested elevations in unusual experiences underpinned the relationship between schizotypy and attenuated LI.^23–27^ However, confounds of learned irrelevance (learning that a US is not contingent upon a CS), conditioned inhibition (CI; learning that a CS predicts the absence of a US), negative priming (reversal of stimuli representing the CS and distractor stimuli) and novel pop-out effects (potentiation of a stimulus’ salience when presented amongst familiar stimuli) limit the strength of conclusions based on LI paradigms.^22^

In addition to LI, tentative evidence suggests that CI (i.e., learning that a CS signals the absence of a US) is also disrupted in high schizotypy individuals and people with schizophrenia. Migo et al. reported that, when presented with a CS, high schizotypy participants exhibited smaller reductions in the expectation of a US in the presence of a conditioned inhibitor.^28^ Also, one study reported that CI was attenuated in a small sample of people with schizophrenia.^29^ However, subsequent studies failed to replicate these findings.^28^^,^^30^

The limitations associated with LI studies highlight the necessity for alterative paradigms to examine the relationship between schizotypy and both LI and CI. Perceptual learning (PL) paradigms may represent a fruitful alternative. PL refers to the phenomenon that pre-exposure to similar stimuli aids subsequent discrimination between them.^31^^,^^32^ McLaren et al. suggested that all stimuli (e.g., “AX” and “BX”) comprise “elements,” including those unique to each stimulus (“A” and “B”) and those common to multiple stimuli (“X”).^33^ Equal pre-exposure to 2 stimuli results in more exposure to, and thus greater decrements in salience (via LI) for, common, compared to unique, elements.^34^ Greater LI of common elements means that a stimulus’ unique elements will more readily become associated with one another than with common elements.^35^ This “unitisation” process promotes generalization between unique elements within stimuli and reduces generalization between unique and common elements, diminishing generalization of learning between AX and BX. Generalization of learning depends on stimuli sharing common elements that have acquired associative strength.^36^ As pre-exposure diminishes the associability of common elements, they will accrue less associative strength, compared to novel stimuli, reducing generalization of learning between stimuli.

The manner of pre-exposure appears important for PL, as pre-exposing stimuli in an intermixed fashion further facilitates discrimination.^37–46^ McLaren et al. argued that this occurs because common elements become independently associated with unique elements in both stimuli during concurrent presentation. During mixed pre-exposure, X is not a reliable predictor of unique elements, meaning each presentation of BX results in the “surprising” absence of A, and vice versa. Meanwhile, repeated presentation of each pair maintains associations between X and both A and B, eventually leading to the formation of an inhibitory association between A and B because they predict the absence of one another.^21^^,^^44–46^ These inhibitory associations further reduce generalization between stimuli, facilitating discrimination. Furthermore, the “associative activation” of absent unique elements has been suggested to increase their salience,^47^ perhaps due to consistently high prediction error,^18^ attenuating any loss of salience through habituation that unique elements would otherwise undergo. In contrast, any inhibitory associations formed following blocked pre-exposure are much weaker and only occur unidirectionally from the unique elements in the second block (B) to those in the first block (A), as the unique presented in the second block (B) will not yet have formed an association with X during initial presentations of AX.

Perceptual learning paradigms represent a useful alternative to LI and CI paradigms. As pre-exposure occurs in the absence of either a US or masking task, learned irrelevance, CI, and negative priming effects do not confound PL paradigms.^22^ Furthermore, the invariance of stimuli between pre-exposure and test phases circumvents problems associated with “novel pop-out effects.” Critically, while the Salience Hypothesis predicts that high schizotypy individuals assign greater salience to redundant stimuli, aberrations in the attribution of salience have previously been indirectly inferred through performance in CS–US contingency learning tasks after pre-exposure. Accordingly, any variability in performance may be attributable to differences in either the pre-exposure or learning phases. In contrast, PL paradigms do not require participants to learn CS–US associations, affording a more direct examination of the attribution of salience to stimuli. Although PL paradigms differ from LI and CI paradigms by not requiring participants to learn CS–US associations, discrimination accuracy, and speed is assumed to rely on similar associative mechanisms.

This study had several aims. The first was to replicate previous demonstrations of PL. Second, this study aimed to examine whether LI and CI are associated with schizotypy dimensions and hallucination proneness (HP). As LI facilitates discrimination following all pre-exposure regimens, but CI facilitates discrimination following only mixed, and not blocked, pre-exposure,^31^^,^^32^ it may be possible to investigate the effect of schizotypy and HP on both LI and CI semi-independently. As research suggests unusual experiences are associated with reduced LI, it was hypothesized that individuals high in these traits would exhibit an attenuation of the facilitatory effect of blocked pre-exposure, relative to novel stimuli. In contrast, if schizotypy is associated with attenuated CI, one would expect a reduction in the facilitatory effect of mixed, relative to blocked, pre-exposure. For transparency, an additional hypothesis concerned the relationship between depressive symptomology (DS) and PL (see Myles^48^); however, space limitations prevent further discussion.

## Methods

### Participants

Consistent with a pre-registered power analysis, 260 adults were recruited using adverts placed on social media and online forums, affording 90% power (https://osf.io/2c6bw/), of which 187, 63, and 9 were female, male, and non-binary, respectively. The age of participants ranged from 18.25 to 82.92 years (*M* = 25.44, SD = 7.54) and all participants received £7.50 following participation. Individuals with schizophrenia^49^^,^^50^ and/or taking antipsychotic medications were ineligible, due to the established impact on LI.^51–57^

### Materials

#### Oxford-Liverpool Inventory of Feelings and Experiences

Schizotypy was measured using the Oxford-Liverpool Inventory of Feelings and Experiences (O-LIFE), a 104 item self-report questionnaire examining unusual experiences, cognitive disorganization, introvertive anhedonia, and impulsive non-conformity.^58^ Support for the reliability of the O-LIFE comes from demonstrations of high internal consistency of each factor^58–60^ and high test–retest reliability over 3-6 months.^61^ Evidence for its validity comes from demonstrations that scores correlate with other relevant measures^62^^,^^63^ and reports that people with schizophrenia score more highly.^64^

#### Multi-Modality Unusual Sensory Experiences Questionnaire

The Multi-Modality Unusual Sensory Experiences Questionnaire (MUSEQ) is a 43-item self-report questionnaire assessing hallucinatory proneness across auditory, visual, olfactory, gustatory, bodily sensations, and sensed presence modalities.^65^ Evidence for the reliability of the MUSEQ comes from reports of high internal consistency and test–retest reliability over 6 months.^65^ Support for its validity comes demonstrations that scores correlate with similar measures^65^ and that individuals with psychosis score more highly.^66^

#### Perceptual Learning Stimuli

**Figure 1:**
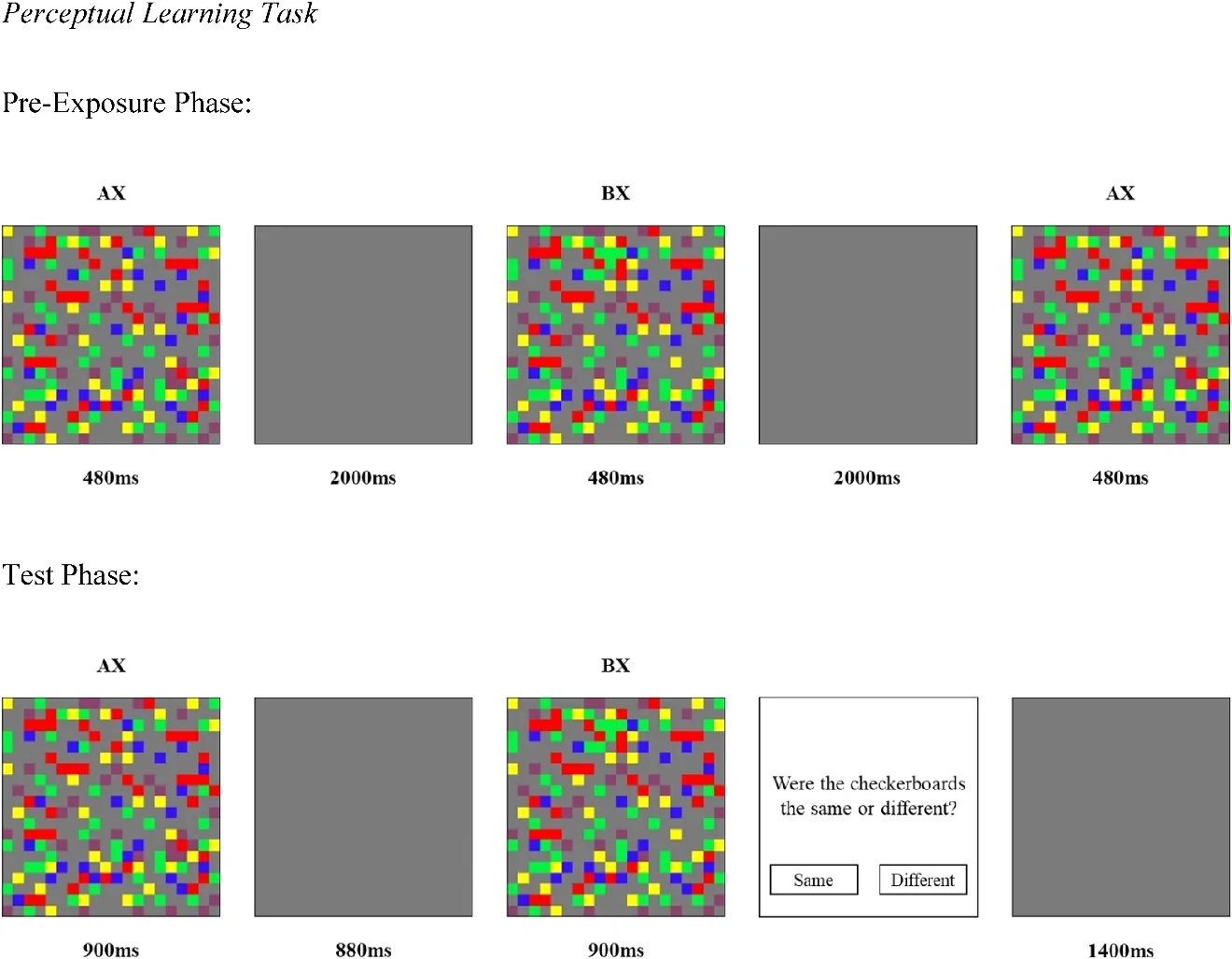
Perceptual Learning Task. The top panel displays the mixed pre-exposure phase, whereas the bottom panel shows the test phase.

Replicating previous PL paradigms,^32^ stimuli consisted of several 20 × 20 checkerboard patterns (see Fig. 1). Common elements were represented by 244 grey squares and 156 squares colored either blue, green, purple, red, or yellow. Unique elements were represented by unique configurations of 6 additional colored squares overlaying squares that would otherwise be grey. Six unique elements were used, denoted by “A,” “B,” “C,” “D,” “E,” and “F,” and 3 common elements were used, denoted by “X,” “Y,” and “Z.” A masking stimulus, consisting of a 20 × 20 checkerboard with only grey squares, segmented stimulus exposures.

### Design and Procedure

Gorilla Experiment Builder was used to create and conduct this study online.^67^ After providing informed consent, participants indicated their age, ethnicity, and gender. A within-subjects design was used, in which all participants completed the O-LIFE, MUSEQ, and PL task.

Following the questionnaires, participants commenced the PL paradigm, depicted in Fig. 1. Consistent with previous studies,^32^^,^^39^^,^^40^^,^^46^ participants were asked to “pay attention to the presented stimuli,” as “any differences detected will be useful later in the experiment.” They were then presented with a series of checkerboards, each preceded by a masking stimulus. Consistent with previous studies,^32^^,^^46^ checkerboards were presented for 480 ms and masking stimuli were presented for 2000 ms. In each condition, participants received exposure to 2 different checkerboards 60 times each (120 total exposures). The pairs of checkerboards were different across these conditions for each participant but were randomized across the sample. Participants were exposed to stimuli in a random order in the “mixed condition.” While traditional PL paradigms presented stimuli in an alternating fashion in the mixed condition, for example,^38^ this pre-exposure regimen was intended to activate analogous associative processes while limiting participants’ opportunity for intentional comparison between stimuli. In the “blocked condition,” they received exposure in 2 separate blocks. The order of mixed and blocked conditions was counterbalanced, as were the stimuli in each condition.

After all pre-exposures, participants completed a “test phase,” in which they were informed that they would “be presented with two checkerboards in succession” and that they “must decide whether the two checkerboards are the same or different.” A 1400 ms masking stimulus was followed by the serial presentation of 2 checkerboards, each of which was presented for 900 ms, separated by an 880 ms masking stimulus. Participants were then required to press a button to indicate whether they thought the stimuli were the same or different.

The test phase consisted of 2 trial types (4 trials in total) presented in a random order. Given that PL effects are largest during early test trials, owing to the reinforcement of correct responding abolishing PL effects once participants become aware of the goal at test, limiting testing to 2 trials was anticipated to produce a larger effect. In the “pre-exposed stimuli trials,” participants determined whether 2 pre-exposed stimuli were the same or different. In the “novel stimuli trials,” they determined whether novel stimuli were the same or different. Participants received 2 of each trial type and were required to respond to proceed to subsequent phases.

### Statistical Analyses

SPSS® 28 was used to analyze the results. All confirmatory analyses were pre-registered (see https://osf.io/2c6bw/). Across all statistical tests, analyses focused on test trials where stimuli were different. Also, for each analysis, participants with mean RTs of greater than 5 s were excluded; a maximum of only 6 cases were excluded for any condition.

To examine PL, a repeated measures ANOVA and^68^ ANOVA examined whether mean reaction times (RTs) and accuracy varied with pre-exposure regimens. For parametric ANOVAs, where^69^ test indicated the assumption of sphericity was violated, the^70^ correction was applied. Bonferroni-corrected paired samples *t*-tests followed up significant results.

Multiple regression using backwards elimination examined whether schizotypy, HP, and DS predicted RTs in the novel stimuli, blocked and mixed pre-exposure conditions, and accuracy in the blocked and mixed pre-exposure conditions. Multinomial logistic regression using backwards elimination examined the effects of schizotypy, HP, and DS on accuracy in the novel stimuli condition. As there were only 2 test trials, participants’ accuracy could only take values of 0%, 50%, and 100% when discriminating between pairs of novel stimuli. Logistic regression models examined the relationship between schizotypy, HP, and DS and which of these (categorical) scores participants were most likely to achieve. Across all regression models, the variable with the smallest effect size in each block was eliminated if its *P*-value was greater than .05.

“Latent inhibition” and “CI” were calculated differently with respect to the dependent variable. For RTs, LI and CI corresponded to RTs in the blocked and mixed pre-exposure conditions, respectively, controlling for RTs in the blocked pre-exposure condition in the latter. For accuracy, LI was calculated by subtracting accuracy scores in the novel stimuli condition from those in the blocked pre-exposure condition, whereas CI was calculated by subtracting accuracy scores in the blocked pre-exposure condition from those in the mixed pre-exposure condition. These equations were used because PL theory suggests that LI underpins increments in performance in the blocked pre-exposure, compared to the novel stimuli, condition and CI enhances performance in the mixed, relative to the blocked, pre-exposure condition.

## Results

### Perceptual Learning

Accuracy scores and RTs were not significantly correlated in any condition (*P* > .1). After applying the Bonferroni correction, age, and gender were not significantly (*P* > .1) correlated with either accuracy or RTs in any condition.

**Figure 2:**
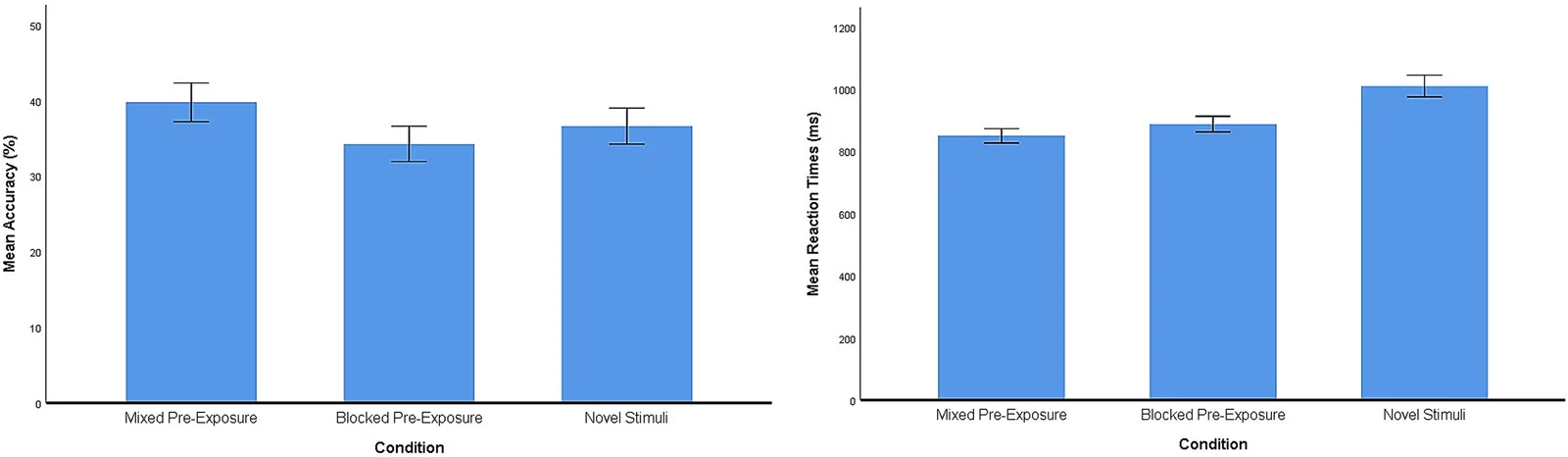
Mean Reaction Times and Accuracy in the Mixed Pre-Exposure, Blocked Pre-Exposure, and Novel Stimuli Conditions. For all bar charts, error bars show ±1 standard error of the mean.

Mean RTs and accuracy scores in each condition are shown in Fig. 2. RTs significantly differed between pre-exposure regimens, F(1.87, 473.14) = 10.743, *P* < .001, η_p_^2^ = .041. Participants discriminated between novel stimuli (*M* = 1010, SD = 559) slower than between stimuli that received either mixed (*M* = 850, SD = 367, *P* < .001) or blocked pre-exposure (*M* = 888, SD = 401, *P* = .006). However, RTs did not significantly differ between conditions (*P* = .695). Accuracy also did not significantly differ between conditions, χ^2^_F_(2) = 1.924, *P* = .382, but was significantly below chance in each condition (*P* < .001).

### Individual Differences and Perceptual Learning

Means, SDs, skewness statistics, and Cronbach’s Alpha^71^ values for each questionnaire dimension are displayed in Table 1. Bonferroni-corrected intercorrelations between questionnaire measures are shown in Table 2. All questionnaire dimensions were significantly correlated (*P* < .01), except for introvertive anhedonia which only correlated with DS scores.

**Table 1:** Means, Standard Deviations, Skewness, and Cronbach’s Alpha Values of the Questionnaires.

Questionnaire	Mean	SD	Skewness	CA
Unusual Experiences (O-LIFE)	9.14	6.16	0.549	0.88
Cognitive Disorganization (O-LIFE)	12.89	6.18	0.011	0.88
Introvertive Anhedonia (O-LIFE)	11.74	2.59	0.320	0.20
Impulsive Non-Conformity (O-LIFE)	11.33	2.60	0.508	0.34
Depressive Symptomology (CES-D)	18.96	11.63	0.602	0.92
Hallucination Proneness (MUSEQ)	40.34	29.25	0.736	0.96

*N* = 259.

**Table 2:** Intercorrelations Between Questionnaire Measures.

Questionnaire	UE	CD	IA	IN	DS	MUSEQ
**UE**	1					
**CD**	0.594^***^	1				
**IA**	0.127	0.125	1			
**IN**	0.492^***^	0.414^***^	0.118	1		
**DS**	0.555^***^	0.702^***^	0.214^**^	0.346^***^	1	
**MUSEQ**	0.671^***^	0.455^***^	−0.003	0.422^***^	0.452^***^	1

*N* = 259. Pearson’s product–moment correlation coefficients between the unusual experiences (UE), cognitive disorganization (CD), introvertive anhedonia (IA), impulsive non-conformity (IN), DS, and MUSEQ scales, alongside Bonferroni-corrected *P*-values.

^**^ *P* < .01. ^***^P < .001.

#### Baseline Discrimination

Unusual experiences, χ^2^(2) = 10.696, *P* = .005, and cognitive disorganization, χ^2^(2) = 8.305, *P* = .016, significantly predicted accuracy in discriminating between novel stimuli, χ^2^(4) = 12.910, *P* = .012, *R*^2^_N_ = .056. More unusual experiences were associated with greater odds of an accuracy of 50%, compared to 0%, *b* = 0.095, Wald χ^2^(1) = 9.960, *P* = .002, OR = 1.099, BCa 95% CI, 0.039-0.167, and marginally greater odds of an accuracy of 50%, compared to 100%, *b* = 0.064, Wald χ^2^(1) = 3.143, *P* = .095, OR = 1.066, BCa 95% CI, −0.013 to 0.154. Also, higher cognitive disorganization scores were associated with significantly lower odds of an accuracy of 50%, compared to both 0%, *b* = −0.076, Wald χ^2^(1) = 6.590, *P* = .01, OR = 0.926, BCa 95% CI, −0.138 to −0.019, and 100%, *b* = −0.082, Wald χ^2^(1) = 4.974, *P* = .026, OR = 0.921, BCa 95% CI, −0.154 to −0.016. However, unusual experiences and cognitive disorganization did not significantly predict the odds of scoring 100%, compared to 0% (*P* > .1).

#### Latent Inhibition

Schizotypy dimensions, HP, and DS did not predict RTs when discriminating between stimuli after blocked pre-exposure. In contrast, unusual experiences and MUSEQ scores significantly predicted accuracy in the LI condition, F(2, 251) = 5.154, *P* = .006. Greater LI was predicted by lower unusual experiences, *β* = −.251, *P* = .005, BCa 95% CI, −0.035 to −0.007, and higher MUSEQ scores, *β* = .240, *P* = .002, BCa 95% CI, 0.001-0.007; similar results were obtained after controlling for RTs in this condition. Importantly, skewness and kurtosis statistics of −0.112 and −0.373, respectively, suggest the residuals approximated a normal distribution.

#### Conditioned Inhibition

Unusual experiences and cognitive disorganization significantly predicted RTs when discriminating between stimuli after mixed pre-exposure, F(2, 255) = 3.329, *P* = .037. Crucially, controlling for RTs in either the novel stimuli or blocked pre-exposure condition produced similar results. These results suggest that greater unusual experiences, *β* = −.163, *P* = .028, BCa 95% CI, −18.841 to −0.723, and lower cognitive disorganization, *β* = .187, *P* = .022, BCa 95% CI, 1.106-19.462, predicted slower RTs in the mixed pre-exposure condition. However, schizotypy dimensions, HP, and DS did not predict accuracy in the CI condition.

## Discussion

This study examined whether high schizotypy individuals assign salience aberrantly and/or exhibit attenuated inhibitory associations, by examining the relationship between PL and both schizotypy and HP. Higher unusual experiences and lower HP scores were associated with attenuated LI, when measured via accuracy. Also, higher unusual experiences and lower cognitive disorganization scores were associated with attenuated CI, when measured via RTs.

### Perceptual Learning

Superior discrimination was observed between pre-exposed, compared to novel, stimuli, consistent with the theory that LI of common elements facilitates discrimination. Consistent with previous studies,^39^^,^^40^ discrimination accuracy was significantly below chance across conditions. This suggests that the high similarity between the stimuli produced a response bias towards reporting that stimuli were “the same.” However, whilst similar studies reported analogous results for accuracy,^32^ the current study only observed this effect for RTs. Several explanations exist for these contradictory findings. First, as Wang and Mitchell did not counterbalance stimuli, the salience of the pre-exposed common or unique elements may have been lower or higher, respectively, than those in the novel stimuli, artificially facilitating discrimination in the pre-exposed group.^72^ Second, Wang and Mitchell presented stimuli in an alternating, rather than random, manner during mixed pre-exposure. Alternating pre-exposure may have afforded greater opportunity for goal-directed comparison of stimuli, inflating the facilitatory effect of mixed, but not blocked, pre-exposure through non-associative learning mechanisms.^73^

Contrasting previous findings^32^^,^^38–40^ and the theory that inhibitory associations between unique elements facilitate discrimination following mixed pre-exposure,^33^^,^^35^ mixed pre-exposure did not produce better discrimination accuracy or RTs than blocked pre-exposure. These discrepant findings may result from differences in stimuli. Using within-subjects designs, previous studies used the same common elements in the mixed (eg, “AX” and “BX”) and blocked (eg, “CX” then “DX”) pre-exposure conditions.^32^^,^^38–40^ During the second pre-exposure phase, prior exposure to X may differentially interfere with performance in each condition. Specifically, after blocked pre-exposure, mixed exposure to X may evoke a representation of D, due to their recent co-occurrence, but is unlikely to evoke a representation of C, as X-C associations likely extinguished during DX exposures. In contrast, after mixed exposure, blocked exposure to X may evoke representations of both A and B, as both recently co-occurred with X. Evoking representations of 2, rather than one, unique elements may cause greater interference of stimulus processing, impeding discrimination after blocked, relative to mixed, pre-exposure. Furthermore, associatively activating 2, rather than one, unique elements may reinstate the salience of the unique elements that received mixed pre-exposure to a greater extent,^47^ facilitating discrimination after mixed pre-exposure. To circumvent these confounds, the current study used different elements in each condition, potentially producing discrepant results.

### Schizotypy, Hallucination Proneness, and Latent Inhibition

Higher unusual experiences and lower HP scores were associated with attenuated LI, as measured by discrimination accuracy. The association between unusual experiences and attenuated LI is consistent with the Salience Hypothesis and prior literature. Elevated stimulus salience may increase the frequency with which spurious associations form between unrelated stimuli,^16–20^^,^^21^^,^^74–76^ which has been argued to culminate in unusual beliefs.^75–77^ Once spurious associations have formed, repeated presentations of stimuli in the *absence* of those (erroneously) expected outcomes will produce prediction errors. As prediction errors can increase stimulus salience,^21^ this may perpetuate the formation of spurious associations between stimuli.^78^

Elevated salience may also contribute to the persistence of unusual beliefs once formed. It has been argued that more salient memories undergo greater consolidation, consequently enhancing memory persistence.^79^ Indeed, evoking memories of a salient CS can strengthen its association with a US, even in the absence of the US,^78^^,^^80^ perhaps because the CS “reminds” participants of the CS–US contingency.^81^ Aberrant stimulus salience may engender more frequent memories, and thus greater consolidation, of erroneous associations in people with schizophrenia.^78^ Also, heightened salience of weakly related, but frequently occurring, stimuli may emulate intermittent reinforcement schedules, producing strong associations between poorly related stimuli.^82^ Thus, elevated salience may cause both the formation and enhanced reconsolidation of unusual beliefs, despite weak *true* associations between stimuli, culminating in persistent unusual beliefs.

The current study was the first to report that HP is associated with potentiated LI. This contradicts the Salience Hypothesis’ proposition that heightened salience underpins both hallucinations and unusual beliefs. Initially, this may seem at odds with the finding that unusual experiences, as measured by the O-LIFE, is associated with attenuated LI. However, whilst past experience of unusual beliefs and hallucinatory experiences is correlated, each can occur in isolation,^83^ suggesting dissociable mechanisms may partially underpin their manifestation. Indeed, whilst the O-LIFE dimension “unusual experiences” comprises unusual beliefs and unusual perceptual experiences,^58^ correlations between scale items and LI suggest that it was specifically items concerning unusual *beliefs* underpinned the effect of unusual experiences on LI (see Supplementary material). This suggests that stimuli retain greater salience in people high in unusual beliefs, rather than unusual perceptual experiences.

As this study was the first to examine the relationship between MUSEQ scores (HP) and LI, failure of previous studies to statistically control for HP, which maintains a strong positive correlation with the O-LIFE’s unusual experiences dimension but an opposing effect on LI, may have occluded the relationship between unusual experiences and LI. Consideration of the nature of the stimuli may allow one to reconcile these results with the Salience Hypothesis. This theory posits that hallucinations reflect the aberrant assignment of salience to “internal” stimuli, such as thoughts. Whilst the Salience Hypothesis lacks a cognitive explanation for this, internal stimuli may be less predictable for high schizotypy individuals, culminating in the accrual of greater salience.^21^ More salient internal stimuli may result in greater attention being directed towards internal stimuli at the expense of external stimuli in the environment,^82^ such as the checkerboards in this study; this may produce greater decrements in salience for external stimuli and consequently potentiated LI. However, this explanation is highly speculative and implies a causal relationship between potentiated LI and HP, which is not substantiated by the current data.

### Schizotypy, Hallucination Proneness, and Conditioned Inhibition

Greater unusual experiences and lower cognitive disorganization scores were associated with attenuated CI, as measured by RTs, consistent with previous demonstrations of attenuated CI in high schizotypy individuals^28^ and people with schizophrenia.^29^ CI is a potential confound in many studies examining the relationship between schizotypy and LI. The current results may indicate that attenuated CI artificially bolstered the relationship between schizotypy and impaired LI in previous studies and/or that prior studies reporting that schizotypy is not associated with LI had designs that were *less* confounded by CI. In other words, attenuated CI in high schizotypy individuals and people with schizophrenia may have exaggerated apparent LI impairments in previous studies.

These results may suggest that impaired CI has a role in unusual experiences. Correlations between scale items and CI indicate that items concerning unusual beliefs, but not unusual perceptual experiences, underpinned the relationship between unusual experiences and CI (see Supplementary material). Reductions in the ability to learn that stimuli predict the absence of outcomes may result in the maintenance of spurious associations, which would otherwise be abolished by CI.^23^ This could produce erroneous beliefs that unrelated events are related, leading to unusual beliefs.^75–77^ The association between unusual experiences and reductions in both LI and CI may also be related. As predictive stimuli often acquire greater salience,^17^^,^^20^ failure to learn that a CS no longer predicts outcomes, due to impaired CI, may maintain the CS’s salience (attenuating LI).^17^^,^^84^ This may cause the manifestation of unusual beliefs through the aforementioned processes.

Moreover, once formed, diminished CI may contribute to the persistence of unusual beliefs. Specifically, impaired CI may reduce an individual’s ability to decrease the perceived strength of CS-US contingencies when USs are omitted upon presentation of CSs. This may attenuate their capacity to “update” erroneous associations, despite contradictory information, producing persistent associations between unrelated stimuli.^75–77^ Evidence for this comes from demonstrations that recollection of extinction is impaired in people with schizophrenia.^85^^,^^86^

### Implications

This study highlights the potential utility of PL paradigms in circumventing confounds present in LI paradigms when evaluating the Salience Hypothesis. Importantly, associations between individual differences and performance were specific to pre-exposure regimens, suggesting that such effects were not the consequence of generalized differences in perceptual discrimination after pre-exposure. Also, the facilitatory effect of unusual experiences and cognitive disorganization on baseline accuracy suggests that associations between impaired PL and both schizotypy and HP are not readily attributable to poorer overall perceptual discrimination. Thus, it is unlikely that these effects are underpinned by confounds that typically burden studies of attention in clinical populations, such as reduced motivation, failure to understand instructions, the sedative effects of medications and cognitive impairments. Moreover, the consistency of these findings with previous literature may indicate that confounds that typically burden LI paradigms entail a relatively small impact on performance in LI paradigms.

### Limitations

Whilst having only 2 test trials per condition minimized the potential for learning during test, it limited the analytical possibilities and the accuracy of parameter estimates. Also, small numbers of test trials may have reduced the appropriateness of using parametric tests to examine the relationship between individual differences and accuracy. Indeed, the ordinal nature of this dependent variable likely lacked sensitivity to variations in LI, potentially occluding relationships between accuracy and individual differences. This lack of statistical power may have obscured PL effects when using accuracy as the dependent variable, precluding fine-grained assessments of discriminability. Relatedly, as the high similarity between stimuli appeared to produce a response bias towards reporting that stimuli were “the same,” there may have been reduced sensitivity to detect subtle individual differences in the effects of schizotypy on PL. This lack of power motivated investigation of the effect of schizotypy and HP on PL, despite null results. Indeed, the consistency of the current findings with those of previous studies on the effect of schizotypy on LI, despite disparate experimental tasks, provides some reassurance that failure to observe PL when using accuracy as the dependent variable may stem from lack of statistical power.

Despite PL theoretically depending on passive exposure, alerting participants to the goal of discriminating between stimuli, by instructing them that “any differences detected (during pre-exposure) will be useful later in the experiment,” potentially reinforced responding to unique elements and diminished responding to common elements.^73^^,^^87^^,^^88^ This may have facilitated discrimination between pre-exposed stimuli and abolished differences in performance following mixed and blocked pre-exposure. However, strategies like comparing whether regions of checkerboards changed across trials failed to reliably determine whether unique elements occupied the respective loci, as participants did not know whether stimuli changed between presentations.

Furthermore, unique elements did not undergo either a summation or so-called retardation test, which are necessary, though not sufficient,^89^ to confirm that stimuli acquired inhibitory properties.^90^ Therefore, unique elements may not have developed reciprocal inhibitory associations during mixed pre-exposure and performance might have been underpinned by other cognitive processes.

Finally, as schizotypy was defined psychometrically, these findings may not generalize to individuals with diagnoses of schizotypal personality disorder, as the latter pertains to some distinct phenomena, such as habitual relational distance, cognitive and perceptual distortions, and eccentricity. Also, as some studies have demonstrated LI is only attenuated in high schizotypy women, but not men,^90^^,^^91^ the predominance of women in the sample may have inflated the effect of schizotypy on LI.

### Future Research

Replication of this study with the outlined improvements is necessary to ensure these results represent robust, reliable findings. Replications should include both clinical and non-clinical samples, to determine whether these findings generalize to people with schizophrenia. Given that high schizotypy participants are by definition “non-clinical,” variations in LI and CI may paradoxically serve as protective factors, preventing these traits from progressing into “schizophrenia.” Thus, contrary to the continuum model,^92–95^ LI and CI may maintain different relationships with positive, negative, and disorganized symptoms in people with schizophrenia. Replicating this study in patients would elucidate the role of these cognitive processes in schizophrenia and may shed light on factors differentiating high schizotypy individuals from those with schizophrenia.

Moreover, future research should evaluate the proposed explanations for the relationships between individual differences and PL. Specifically, studies must determine whether variations in LI and CI maintain a causal role in schizotypy and schizophrenia. Also, future research may benefit from controlling for sex, smoking, and cannabis use, given their known effects on LI.^96^ Furthermore, researchers should investigate the factors causing alterations to LI and CI, examining the causal pathways between biopsychosocial risk factors, alterations to learning mechanisms and both schizotypy and HP.

## Conclusions

In conclusion, the results tentatively suggested that unusual experiences were associated with attenuated LI, suggesting that aberrant salience may underpin the development of unusual beliefs. Paradoxically, this study ascertained provisional evidence that HP was associated with potentiated LI, perhaps suggesting the salience of redundant stimuli declines faster in hallucination-prone individuals. Finally, this study tentatively indicated that unusual beliefs and cognitive disorganization are associated with attenuated and potentiated CI, respectively. Nevertheless, as this study did not replicate some previous PL effects, namely that mixed pre-exposure leads to superior discrimination accuracy than blocked pre-exposure, the current findings should be treated as preliminary. Further studies with greater statistical power are required to validate these results.

## Supplementary Material

Supplementary_Materials_v5_sgag031
