# Supplementary material for: The Relationship Between Schizotypy and Latent Inhibition and Conditioned Inhibition
Source: Schizophr Bull Open. 2026 Jul 31;7(1):sgag031. doi: 10.1093/schizbullopen/sgag031 (PMC13489161; doi:10.1093/schizbullopen/sgag031)
Supplement: Supplementary file 1 [file supplementary_materials_v5_sgag031.docx]

# Appendices Contents

**Appendix 1: Results Section Regression Models**

**Appendix 2: Gender Identity and Ethnicity of Participants**

**Appendix 3: Materials**

**Appendix 4: Visual Perceptual Learning Stimuli**

**Appendix 5: Statistical Analyses**

**Appendix 6: Additional Analyses**

**Appendix 7: Intercorrelations Between Scale Items and Performance**

**Appendix 8: Aberrant Salience and Depression**

**References**

## **Appendix 1: Results Section Regression Models**

**Table 4**

Regression Models Show No Effect of Depressive Symptomology, Schizotypy Dimensions or Hallucination Proneness on Reaction Times in the Blocked Pre-Exposure Condition

| **Model** | **R** | **R^2^** | **Adjusted R^2^** | **DW** | **ANOVA Statistics** | **Variable with Smallest Effect** | **Standardised β Coefficient** | **Variable Significance** |
| --- | --- | --- | --- | --- | --- | --- | --- | --- |
| **1** | .104 | .011 | -.013 | 1.879 | F(6, 249) = .452,  p = .843 | MUSEQ | -.015 | .862 |
| **2** | .103 | .011 | -.009 | 1.881 | F(5, 250) = .539,  p = .747 | Impulsive Non-Conformity | .026 | .726 |
| **3** | .101 | .010 | -.006 | 1.878 | F(4, 251) = .645,  p = .631 | Unusual Experiences | -.024 | .762 |
| **4** | .099 | .010 | -.002 | 1.875 | F(3, 252) = .832,  p = .477 | Cognitive Disorganisation | -.079 | .371 |
| **5** | .082 | .007 | -.001 | 1.875 | F(2, 253) = .847,  p = .430 | CES-D | .051 | .424 |
| **6** | .064 | .004 | < .001 | 1.879 | F(1, 254) = 1.053,  p = .306 | - | - | - |

*Note.* N = 256. The variables entered into each model are displayed below.

1. CES-D, Unusual Experiences, Cognitive Disorganisation, Introvertive Anhedonia, Impulsive Non-Conformity and MUSEQ
2. CES-D, Unusual Experiences, Cognitive Disorganisation, Introvertive Anhedonia and Impulsive Non-Conformity
3. CES-D, Unusual Experiences, Cognitive Disorganisation and Introvertive Anhedonia
4. CES-D, Cognitive Disorganisation and Introvertive Anhedonia
5. CES-D and Introvertive Anhedonia
6. Introvertive Anhedonia

**Table 5**

Regression Models Showing Unusual Experiences and Hallucination Proneness Predicts Latent Inhibition (Accuracy)

| **Model** | **R** | **R^2^** | **Adjusted R^2^** | **DW** | **ANOVA Statistics** | **Variable with Smallest Effect** | **Standardised β Coefficient** | **Variable Significance** |
| --- | --- | --- | --- | --- | --- | --- | --- | --- |
| **1** | .228 | .052 | .029 | 1.938 | F(6, 247) = 2.265,  p = .038 | Introvertive Anhedonia | -.025 | p = .737,  [-.035, .026] |
| **2** | .227 | .052 | .032 | 1.939 | F(5, 248) = 2.697,  p = .021 | Impulsive Non-Conformity | .059 | p = .447,  [-.019, .042] |
| **3** | .221 | .049 | .034 | 1.927 | F(4, 249) = 3.211,  p = .014 | Cognitive Disorganisation | .097 | p = .285,  [-.007, .023] |
| **4** | .212 | .045 | .033 | 1.934 | F(3, 250) = 3.914,  p = .009 | CES-D | -.089 | p = .172,  [-.010, .001] |
| **5** | .199 | .039 | .032 | 1.939 | F(2, 251) = 5.154,  p = .006 | - | - | - |

*Note.* N = 254. The variables entered into each model are displayed below. For models examining the effect of individual differences on accuracy in the LI and CI conditions, BCa 95% CI based on 1000 bootstrapped samples are reported in brackets under ‘Variable Significance.’

1. CES-D, Unusual Experiences, Cognitive Disorganisation, Introvertive Anhedonia, Impulsive Non-Conformity and MUSEQ
2. CES-D, Unusual Experiences, Cognitive Disorganisation, Impulsive Non-Conformity and MUSEQ
3. CES-D, Unusual Experiences, Cognitive Disorganisation and MUSEQ
4. CES-D, Unusual Experiences and MUSEQ
5. Unusual Experiences and MUSEQ

**Table 6**

Regression Models Showing Unusual Experiences and Cognitive Disorganisation Predict Reaction Times in the Mixed Pre-Exposure Condition

| **Model** | **R** | **R^2^** | **Adjusted R^2^** | **DW** | **ANOVA Statistics** | **Variable with Smallest Effect** | **Standardised β Coefficient** | **Variable Significance** |
| --- | --- | --- | --- | --- | --- | --- | --- | --- |
| **1** | .183 | .034 | .011 | 1.958 | F(6, 251) = 1.456,  p = .194 | Impulsive Non-Conformity | .006 | .937 |
| **2** | .183 | .034 | .014 | 1.958 | F(5, 252) = 1.753,  p = .123 | MUSEQ | -.062 | .465 |
| **3** | .178 | .032 | .016 | 1.957 | F(4, 253) = 2.062,  p = .086 | Introvertive Anhedonia | -.052 | .408 |
| **4** | .170 | .029 | .017 | 1.965 | F(3, 254) = 2.523,  p = .058 | CES-D | .085 | .340 |
| **5** | .160 | .025 | .018 | 1.970 | F(2, 255) = 3.329,  p = .037 | - | - | - |

*Note.* N = 258. The variables entered into each model are displayed below.

1. CES-D, Unusual Experiences, Cognitive Disorganisation, Introvertive Anhedonia, Impulsive Non-Conformity and MUSEQ
2. CES-D, Unusual Experiences, Cognitive Disorganisation, Introvertive Anhedonia and MUSEQ
3. CES-D, Unusual Experiences, Cognitive Disorganisation and Introvertive Anhedonia
4. CES-D, Unusual Experiences and Cognitive Disorganisation
5. Unusual Experiences and Cognitive Disorganisation

**Table 7**

Regression Models Show No Effect of Depressive Symptomology, Schizotypy Dimensions or Hallucination Proneness on Conditioned Inhibition (Accuracy)

| **Model** | **R** | **R^2^** | **Adjusted R^2^** | **DW** | **ANOVA Statistics** | **Variable with Smallest Effect** | **Standardised β Coefficient** | **Variable Significance** |
| --- | --- | --- | --- | --- | --- | --- | --- | --- |
| **1** | .130 | .017 | -.007 | 1.917 | F(6, 248) = .711,  p = .641 | Introvertive Anhedonia | .011 | p = .877,  [-.028, .033] |
| **2** | .130 | .017 | -.003 | 1.917 | F(5, 249) = .851,  p = .515 | Unusual Experiences | .065 | p = .456,  [-.009, .020] |
| **3** | .123 | .015 | -.001 | 1.901 | F(4, 250) = .954,  p = .433 | MUSEQ | -.071 | p = .322,  [-.004, .001] |
| **4** | .107 | .011 | < .001 | 1.880 | F(3, 251) = .968,  p = .408 | Impulsive Non-Conformity | .078 | p = .253,  [-.013, .042] |
| **5** | .080 | .006 | -.001 | 1.869 | F(2, 252) = .811,  p = .446 | CES-D | .104 | p = .212,  [-.003, .012] |
| **6** | .031 | .001 | -.003 | 1.864 | F(1, 253) = .237,  p = .627 | - | - | - |

*Note.* N = 255. The variables entered into each model are displayed below.

1. CES-D, Unusual Experiences, Cognitive Disorganisation, Introvertive Anhedonia, Impulsive Non-Conformity and MUSEQ
2. CES-D, Unusual Experiences, Cognitive Disorganisation, Impulsive Non-Conformity and MUSEQ
3. CES-D, Cognitive Disorganisation, Impulsive Non-Conformity and MUSEQ
4. CES-D, Cognitive Disorganisation and Impulsive Non-Conformity
5. CES-D and Cognitive Disorganisation
6. Cognitive Disorganisation

## **Appendix 2: Gender Identity and Ethnicity of Participants**

**Table 10**

*Gender Identity and Ethnicity of Participants*

| **Gender Identity** | **Percentage of Sample** | **Number of Participants** |
| --- | --- | --- |
| Female | 71.9% | 187 |
| Gender Fluid | .4% | 1 |
| Male | 24.2% | 63 |
| Non-Binary | 3.1% | 8 |
| Other | .4% | 1 |
| **Ethnicity** | **Percentage of Sample** | **Number of Participants** |
| African | .8% | 2 |
| Arab | 1.9% | 5 |
| Asian | 1.9% | 5 |
| Asian & Hispanic | .4% | 1 |
| British | 46.2% | 120 |
| Chinese | 8.5% | 22 |
| Hispanic | 2.3% | 6 |
| Indian | 9.2% | 24 |
| Indonesian | .4% | 1 |
| Iranian | .4% | 1 |
| Irish | 1.9% | 5 |
| Japanese | .4% | 1 |
| Other Ethnic Group | .8% | 2 |
| Pakistani | 1.9% | 5 |
| Slavic | .4% | 1 |
| Turkish | .8% | 2 |
| White (Non-British) | 16.2% | 42 |
| White and Asian | 3.5% | 9 |
| White and Black African | 1.5% | 4 |
| White and Black Caribbean | .8% | 2 |

## **Appendix 3: Materials**

Gorilla Experiment Builder was used to create and conduct this study online (Anwyl-Irvine et al., 2020). All of the materials are detailed below and freely available online (see <https://app.gorilla.sc/openmaterials/434012>).

*Bot Check:*

A bot check prevented bots from producing spurious data. Participants were presented with grids of 25 numbers; each cell contained either a ‘1’ or ‘0.’ Across two puzzles, participants had to report the number of times ‘0’ was presented. They were given three attempts at each puzzle. If participants failed three times, they had to wait 30 seconds before re-attempting.

*Oxford-Liverpool Inventory of Feelings and Experiences:*

Schizotypy was measured using the O-LIFE, a 104 item self-report questionnaire examining unusual experiences, cognitive disorganisation, introvertive anhedonia and impulsive non-conformity (Mason et al., 1995). Participants responded to questions on a dichotomous scale, consisting of ‘Yes’ and ‘No.’ ‘Unusual experiences,’ examined with 30 items, refer to unusual perceptual experiences and beliefs. For example, one item asked, “Do you believe in telepathy?” ‘Cognitive disorganisation,’ measured by 24 items, pertains to difficulties with attention, concentration, decision making, emotional lability and anxiety. For example, one item asked, “Is it hard for you to make decisions?” ‘Introvertive anhedonia,’ assessed using 27 items, corresponds to intimacy avoidance and loss of pleasure. For example, one item asked, “Do you prefer watching television to going out with people?” Finally, ‘impulsive non-conformity,’ measured with 23 items, pertains to reckless, self-abusive and violent behaviour. For example, one item asked, “Do you often have an urge to hit someone?”

The factor structure of the O-LIFE has ascertained robust support. Research suggests the factors maintain high internal consistency (Mason, 1995; Mason & Claridge, 2006; Mason, 2015; Vollema & Bosch, 1995), with the exception of impulsive non-conformity (Lin et al., 2013), and scores are relatively stable across time, with high test-retest reliability over intervals of 3-6 months (Burch et al., 1998). Support for the construct validity of the O-LIFE comes from demonstrations that scores correlate with other relevant measures, such as dissociative experiences, childhood abuse and mental health difficulties (Goulding, 2004; Startup, 1999). Moreover, individuals with schizophrenia score higher in unusual experiences, cognitive disorganisation and introvertive anhedonia than neurotypicals (Cochrane et al., 2010), highlighting the relevance of these scales to schizophrenia. Therefore, the O-LIFE was considered both a valid and reliable measure of schizotypy.

*Centre for Epidemiologic Studies Depression Scale:*

Depressive symptomology was examined using the Centre for Epidemiologic Studies Depression Scale (CES-D), a 20-item self-report questionnaire assessing the frequency with which individuals experienced DS over the preceding week (Radloff, 1977). Participants rated their agreement with statements on a four-point Likert scale, consisting of ‘Rarely or None of the Time,’ ‘Some or a Little of the Time,’ ‘Occasionally or a Moderate Amount of Time’ and ‘Most or All of the Time.’ For example, one item stated, “I felt lonely.”

Evidence for the construct validity of the CES-D comes from repeated demonstrations that individuals with depression exhibit higher scores than neurotypicals (Radloff, 1977); scores concur with clinicians’ evaluations of patients, suggesting this scale represents a relatively unbiased measure (Craig & Natta, 1976). Indeed, scores correlate with other theoretically relevant measures, including Beck’s Hopelessness Scale (Yang et al., 2015), Beck’s Depression Inventory-II (Lipps et al., 2007; Segal et al., 2008; Skorikov & VanderVoort, 2003; Wang & Gorenstein, 2013; Yang et al., 2015), life events and therapeutic interventions (Radloff, 1977). Demonstrations that the CES-D exhibits high sensitivity and specificity, pertaining to the correct identification of people with and without depression, respectively, across several demographic groups provides further support for its validity (González-Roz et al., 2019; Joode et al., 2019; Vilagut et al., 2016). Also, this scale maintains high internal reliability across several cultures (Cosco et al., 2017; Jiang et al., 2019; Skorikov & VanderVoort, 2003) and a range of DS severities (Ballegooijen et al., 2016; Dijk et al., 2018; Yang et al., 2015). Thus, the CES-D was considered both a valid and reliable measure of DS.

*Multi-Modality Unusual Sensory Experiences Questionnaire:*

Unusual sensory experiences are discrepancies between one’s perception and reality, and occur throughout the general population (McGrath et al., 2015; Nuevo et al., 2012; Pechey & Halligan, 2012). The Multi-Modality Unusual Sensory Experiences Questionnaire (MUSEQ) is a 43-item self-report questionnaire assessing USEs across six modalities, including the auditory, visual, olfactory, gustatory, bodily sensations and sensed presence modalities (Mitchell et al., 2017). Participants indicated their agreement with statements, each of which corresponds to a USE of varying severity, on a five-point Likert scale, consisting of ‘Never,’ ‘Hardly Ever,’ ‘Rarely,’ ‘Occasionally’ and ‘Frequently.’ An example of a ‘subclinical auditory’ item is, “My ears have played tricks on me,” while “I have heard a person’s voice and then found that no-one was there” is an ‘auditory hallucination’ item.

Factor analytic studies suggest that USEs in each modality are independent but related to an overarching factor termed ‘hallucination proneness’ (HP; Mitchell et al., 2017). These factors maintain high internal consistency and acceptable test-retest reliability over six-month intervals (Mitchell et al., 2017). Evidence for the convergent validity of this scale comes from demonstrations that scores correlate with similar measures, including the Cardiff Anomalous Perception Scale (Bell et al., 2006), LSHS-Modified-II (Larøi & Linden, 2005) and unusual experiences dimension of the O-LIFE (Mitchell et al., 2017). Importantly, the MUSEQ maintained strong discriminant validity, as scores did not correlate with cognitive disorganisation or introvertive anhedonia. Furthermore, the scale’s relevance to schizophrenia is supported by reports that individuals with psychosis display elevated scores (Chouinard et al., 2019). Therefore, the MUSEQ was considered both a valid and reliable measure of HP.

*Visual Perceptual Learning Stimuli:*

Replicating previous PL paradigms (Wang & Mitchell, 2011), visual stimuli consisted of several 20 x 20 checkerboard patterns, created using Microsoft Excel (see **Appendix 4**). Sample stimuli are displayed in **Figure 3**. Each checkerboard contained 244 grey squares and 156 squares coloured either blue, green, purple, red or yellow; common elements were represented by these coloured squares. Unique elements were represented by unique configurations of six additional coloured squares overlaying squares that would otherwise be grey. Six unique elements were used, denoted by ‘A,’ ‘B,’ ‘C,’ ‘D,’ ‘E’ and ‘F,’ and three common elements were used, denoted by ‘X,’ ‘Y’ and ‘Z.’ Each stimulus consisted of a combination of one unique element and one common element. The ‘A,’ ‘B,’ ‘C,’ ‘D,’ ‘X’ and ‘Y’ elements were identical to those used in previous studies. However, ‘C’ was altered, as one of the six squares overlayed a coloured square in X; this square was replaced with another coloured square that was occupied by a grey square across all stimuli. Also, a masking stimulus, consisting of a 20 x 20 checkerboard with only grey squares, segmented stimulus exposures.

**Figure 3**

*Sample Stimuli*

*Note.* AX is shown on the left and BX is shown on the right. Unique elements are surrounded by a black border; however, this was not present in the experiment. The remaining coloured squares correspond to the common element.

To counterbalance stimuli, the author created two additional unique elements, denoted by ‘E’ and ‘F,’ and one additional common element, denoted by ‘Z;’ creation of these stimuli was guided by procedures used in previous studies (Lavis & Mitchell, 2006). To create ‘Z,’ 156 coloured squares were randomly assigned to loci across the checkerboard, with the pre-requisition that grey squares must occupy the loci of all unique elements; this enabled the combination of every unique element with each common element. The location of ‘E’ and ‘F’ was determined by the availability of clusters of adjacent grey squares across all common elements. Furthermore, the proportion of blue, green, purple, red and yellow squares in each element was randomly determined.

## **Appendix 4: Perceptual Learning Stimuli**

| **Stimulus Name** | **Stimulus** | **Stimulus Name** | **Stimulus** |
| --- | --- | --- | --- |
|  |  |  |  |
| AX |  | DY |  |
| AY |  | DZ |  |
| AZ |  | EX |  |
| BX |  | EY |  |
| BY |  | EZ |  |
| BZ |  | FX |  |
| CX |  | FY |  |
|  |  |  |  |

| **Stimulus Name** | **Stimulus** | **Stimulus Name** | **Stimulus** |
| --- | --- | --- | --- |
|  |  |  |  |
| CY |  | FZ |  |
| CZ |  | Masking Stimulus |  |
| DX |  |  |  |
|  |  |  |  |

**Appendix 5: Statistical Analyses**

All confirmatory analyses were pre-registered (see <https://osf.io/2c6bw/>). Whilst the author initially intended to use accuracy scores and RTs in each condition as indicators for a latent variable representing multiple facets of ‘performance,’ weak correlations between accuracy scores and RTs (p > .1) prevented this. Therefore, accuracy and RTs were analysed separately.

*Managing Outliers and Missing Data:*

As extreme values can bias parameter estimates and the sum of squared errors, participants completing trials in an atypical manner were excluded. Whilst defining ‘atypical’ is subjective, a mean RT of greater than five seconds was considered indicative of an atypical strategy in the respective condition. Participants were only excluded from analyses involving conditions in which their mean RT exceeded five seconds, and not from those in which their mean RT for all relevant conditions fell within this limit; this threshold resulted in a maximum of only six cases being excluded for any condition. Furthermore, due to the paucity of missing questionnaire data, missing values were estimated. The procedures used to estimate missing data are detailed in **Appendix 6**, **Additional Analysis 5**.

*Normality:*

Central Limit Theorem suggests that sampling distributions of parameter estimates will approximate normal distributions in large samples (Field, 2013). As this study had a relatively large sample, data were assumed to approximate normal distributions across all parametric analyses; inspection of histograms showing the distribution of residuals corroborated this assumption. To analyse the effect of pre-exposure condition on accuracy and of individual differences on accuracy in discriminating between novel stimuli, statistical tests that do not assume normality were used because mean accuracy scores could only take values of 0%, 50% and 100%, and therefore represented an ordinal variable.

*Perceptual Learning:*

One-way repeated measures ANOVAs examined whether mean RTs in discriminating between stimuli varied with respect to whether participants received mixed, blocked or no pre-exposure. Provisional checks for bias examined the data for sphericity (Mauchly, 1940). Post-hoc paired samples t-tests using the Bonferroni correction followed up significant (p < .05) omnibus tests.

Friedman's (1937) ANOVAs (χ^2^_F_) examined whether mean accuracy in discriminating between stimuli varied with respect to whether participants received mixed, blocked or no pre-exposure. Post-hoc Wilcoxon (1945) matched-pair signed-rank tests using the Bonferroni correction followed up significant (p < .05) main effects. Also, Bonferroni-corrected Wilcoxon signed-rank tests examined whether accuracy significantly differed from chance (50%) in the PL tasks.

*Individual Differences and Perceptual Learning:*

Multiple regression using backwards elimination examined whether schizotypy dimensions, HP and DS predicted RTs in the novel stimuli, LI and CI conditions and accuracy in the LI and CI conditions. As the length of the dependent variable was ordinal in analyses concerning accuracy scores, capable of taking five values, bias corrected and accelerated 95% confidence intervals (BCa 95% CI) based on 1000 bootstrapped samples were reported across all models, producing accurate confidence intervals. Also, skewness and kurtosis statistics were reported to corroborate claims of normally distributed residuals. In each block, the variable with the smallest effect size was eliminated if its p-value was greater than .05; variables were not eliminated if deletion significantly (p < .05) detracted from the model’s ability to predict the outcome. Across all analyses, when theoretically relevant variables were eliminated, forced entry regression analyses examined the independent effect of these variables. Provisional checks for bias were conducted for each analysis. Across all analyses, Durbin-Watson (DW; 1951) test statistics were greater than 1 and less than 3, suggesting the residuals were independent (Field, 2013). Also, the assumptions of homoscedasticity and linearity, examined through inspection of plots of the predicted values against the residual values, were met across all models. Finally, multicollinearity was not considered problematic in any analysis, as all tolerance statistics were above .20 (Menard, 2002).

Multinomial logistic regression using backwards elimination examined the effects of schizotypy dimensions, HP and DS on accuracy in the novel stimuli condition. In each block, the variable with the smallest effect size, based on the likelihood ratio, was eliminated if its p-value was greater than .05; variables were not eliminated if deletion significantly (p < .05) detracted from the model’s ability to predict the outcome. Multicollinearity was not considered problematic in any analysis, as all tolerance statistics were above .20 (Menard, 2002). Also, the assumption of linearity was met across all analyses, as indicated by the non-significant (p > .05) contribution of the interaction between predictors and the log of the respective predictors to each model (Field, 2013).

## **Appendix 6: Additional Analyses**

In accordance with the philosophy of ‘multi-versing’ (Steegen et al., 2016), the data were analysed in a multitude of ways, to increase the transparency of analytic decisions and provide a holistic overview of the data.

*Additional Analysis 1 – Visual Pre-Exposed Common Elements with Novel Unique Elements Excluding Outliers (Reaction Times)*

A one-way repeated measures ANOVA examined whether reaction times in discriminating between similar visual stimuli varied between the mixed pre-exposed common elements with novel unique elements, blocked pre-exposed common elements with novel unique elements and novel stimuli conditions. Eight outliers were removed, resulting in 252 cases. The assumption of sphericity was met for the main effect of pre-exposure condition, χ^2^(2) = 2.702, p = .259.

After these provisional analyses, the main effect was examined. **Figure 4** details participants’ mean reaction times in each condition. The results indicate that reaction times significantly differed between pre-exposure conditions, F(2, 502) = 3.313, p = .037, η_p_^2^ = .013; this represents a small effect size (Lakens, 2013). Post-hoc pairwise comparisons using the Bonferroni correction indicated that participants exhibited significantly faster reaction times in the blocked pre-exposed common elements with novel unique elements (M = 893, SD = 470), compared to the novel stimuli (M = 1001, SD = 541), condition (p = .023). However, reaction times did not significantly differ between the mixed pre-exposed common elements with novel unique elements condition (M = 935, SD = 538) and either the blocked pre-exposed common elements with novel unique elements condition (p = .987) or the novel stimuli condition (p = .398).

**Figure 4**

Mean Reaction Times in the Visual Mixed Pre-Exposed Common Elements with Novel Unique Elements, Blocked Pre-Exposed Common Elements with Novel Unique Elements and Novel Stimuli Conditions

*Note.* N = 252. Reaction times are reported in milliseconds. Error bars show +/- 1 standard error of the mean.

*Additional Analysis 2 – Visual Pre-Exposed Common Elements with Novel Unique Elements Excluding Outliers (Accuracy):*

Friedman’s ANOVA examined whether accuracy in discriminating between visual stimuli varied between the mixed pre-exposed common elements with novel unique elements, blocked pre-exposed common elements with novel unique elements and novel stimuli conditions. Eight outliers were removed, resulting in 252 cases. The results indicated that accuracy significantly differed with respect to pre-exposure condition, χ^2^_F_(2) = 6.434, p = .04.

**Figure** **5** details participants’ mean accuracy in each condition. Post-hoc Wilcoxon (1945) signed-rank tests using the Bonferroni correction indicated that participants exhibited significantly higher accuracy in the blocked pre-exposed common elements with novel unique elements, compared to the novel stimuli, condition, T = 4564.5, p = .03, r = .11**;** this represents a small effect size (Field, 2013). However, accuracy did not significantly differ between either the mixed and blocked pre-exposed common elements with novel unique elements conditions, T = 6142.5, p = .81, or the mixed pre-exposed common elements with novel unique elements and the novel stimuli conditions, T = 5945, p = .46.

**Figure 5**

Mean Accuracy in the Visual Mixed Pre-Exposed Common Elements with Novel Unique Elements, Blocked Pre-Exposed Common Elements with Novel Unique Elements and Novel Stimuli Conditions

*Note.* N = 252. Error bars show +/- 1 standard error of the mean.

*Additional Analysis 3 – Visual Pre-Exposed Unique Elements with Novel Common Elements Excluding Outliers (Reaction Time):*

A one-way repeated measures ANOVA examined whether reaction times in discriminating between similar visual stimuli varied between the mixed pre-exposed unique elements with novel common elements, blocked pre-exposed unique elements with novel common elements and novel stimuli conditions. Five outliers were removed, resulting in 255 cases. The assumption of sphericity was met for the main effect of pre-exposure condition, χ^2^(2) = 4.911, p = .086.

After these provisional analyses, the main effect was examined. **Figure 6** details participants’ mean reaction times in each condition. The results indicate that reaction times significantly differed with respect to pre-exposure condition, F(2, 508) = 6.255, p = .002, η_p_^2^ = .024; this represents a small effect size (Lakens, 2013). Post-hoc pairwise comparisons using the Bonferroni correction indicated that participants exhibited significantly faster reaction times in the mixed pre-exposed unique elements with novel common elements (M = 879, SD = 518), compared to the novel stimuli (M = 1012, SD = 559), condition (p = .008) and in the blocked pre-exposed unique elements with novel common elements (M = 893, SD = 469), compared to the novel stimuli, condition (p = .009). However, reaction times did not significantly differ between the mixed and blocked pre-exposed unique elements with novel common elements conditions (p = 1).

**Figure 6**

Mean Reaction Times in the Visual Mixed Pre-Exposed Unique Elements with Novel Common Elements, Blocked Pre-Exposed Unique Elements with Novel Common Elements and Novel Stimuli Conditions

*Note.* N = 255. Reaction times are reported in milliseconds. Error bars show +/- 1 standard error of the mean.

*Additional Analysis 4 – Visual Pre-Exposed Unique Elements with Novel Common Elements Excluding Outliers (Accuracy):*

Friedman’s ANOVA examined whether accuracy in discriminating between visual stimuli varied between the mixed pre-exposed unique elements with novel common elements, blocked pre-exposed unique elements with novel common elements and novel stimuli conditions. Five outliers were removed, resulting in 255 cases. The results indicated that accuracy significantly differed with respect to pre-exposure condition, χ^2^_F_(2) = 16.599, p < .001.

**Figure** **7** details participants’ mean accuracy in each condition. Post-hoc Wilcoxon (1945) signed-rank tests using the Bonferroni correction indicated that participants exhibited significantly higher accuracy in the mixed pre-exposed unique elements with novel common elements, compared to the novel stimuli, condition, T = 3810, p < .001, r = .18; this represents a small effect size (Field, 2013). Also, participants exhibited marginally higher accuracy in the blocked pre-exposed unique elements with novel common elements, compared to the novel stimuli, condition, T = 4738.5, p = .06. However, accuracy did not significantly differ between the mixed and blocked pre-exposed unique elements with novel common elements conditions, T = 5092.5, p = .22.

**Figure 7**

Mean Accuracy in the Visual Mixed Pre-Exposed Unique Elements with Novel Common Elements, Blocked Pre-Exposed Unique Elements with Novel Common Elements and Novel Stimuli Conditions

*Note.* N = 255. Error bars show +/- 1 standard error of the mean.

*Additional Analysis 5 – Estimating Missing Data:*

**Table** **11** details the mean number of items missed by participants with missing data for each questionnaire dimension. Due to the paucity of missing data, missing values were estimated. Indeed, with the exception of one individual, participants omitted a maximum of one item on each questionnaire dimension; excluding all of a participant’s data due to one omission would be inappropriate and would notably reduce statistical power. However, one participant omitted three items from the CES-D, four items from the impulsive non-conformity dimension of the O-LIFE and one item from the olfactory subscale of the MUSEQ; this participant was excluded from all analyses using these measures, due to large amounts of missing data.

**Table 11**

Number of Participants with Missing Data and Mean Number of Items Missed

| **Questionnaire** | **Number of Participants with Missing Data** | **Mean Number of Missing Items** |
| --- | --- | --- |
| O-LIFE – Unusual Experiences | 6 | 1 |
| O-LIFE – Cognitive Disorganisation | 6 | 1 |
| O-LIFE – Introvertive Anhedonia | 4 | 1 |
| O-LIFE – Impulsive Non-Conformity | 7 | 1.4 |
| CES-D | 10 | 1.2 |
| MUSEQ – Auditory | 1 | 1 |
| MUSEQ – Visual | 3 | 1 |
| MUSEQ – Olfactory | 5 | 1 |
| MUSEQ – Gustatory | 2 | 1 |
| MUSEQ – Bodily Sensation | 4 | 1 |
| MUSEQ – Sensed Presence | 2 | 1 |

*Note.* The lefthand column details the relevant questionnaire dimension. In the central column, the number of participants that omitted at least one item on the respective questionnaire are displayed. In the righthand column, the mean number of items missed by participants that omitted at least one item on the respective dimension is reported.

Multiple regression was used to estimate missing values. Specifically, subscale items, excluding the item with missing data, were used to predict the overall scale score, which was calculated by summating the values constituting the respective subscale. For example, if a participant failed to respond to item five on a four-item scale, then a regression model to predict the overall scale score would be generated based on the first four items. The value of the missing item was then estimated using the difference between the predicted overall scale score and the value of the summated scores from the available data. For example, if the multiple regression model predicted that a participant’s score on the fictitious five-item scale would be 10, but the values from the first four items summated to 8, then the participant’s score on item 5 would be estimated to be 2.

Separate forced entry multiple regression models were calculated for each item with missing data. Provisional checks for bias in the data were conducted. Firstly, the assumption that the dependent variable was linearly related to predictors was met, as the dependent variable, by definition, was the product of the summation of the predictors. Secondly, outliers were not removed, to limit the risk of overfitting. Thirdly, the data were assumed to approximate a normal distribution, on the basis of Central Limit Theorem (Field, 2013). Fourthly, across all models, Durbin-Watson (1951) test statistics were close to 2, indicating that the assumption of independent errors was met. Fifthly, across all models, the assumption of homoscedasticity was met, as indicated by inspection of plots of the predicted values against the residual values. Finally, multicollinearity was not considered to be problematic in any model, as all tolerance statistics were above .2 (Menard, 2002). For parsimony, the equations used to estimate missing data have not been included in this appendix.

*Additional Analysis 6 – Individual Differences and Visual Blocked Pre-Exposure (Accuracy):*

Multinomial logistic regression using backwards deletion examined the effects of schizotypy, HP and DS on accuracy in the visual blocked pre-exposure condition. Exclusion of outliers resulted in 256 participants. **Table 12** details the iterations of the regression models. Across all models, multicollinearity was not considered to be problematic, as all tolerance statistics were above .2 (Menard, 2002). Also, the assumption of linearity was met across all models, as indicated by the non-significant (p > .05) contribution of the interaction between predictors and the log of the respective predictors to each model (Field, 2013).

The final model indicated that MUSEQ scores significantly predicted accuracy, χ^2^(2) = 10.688, p = .005, R^2^_N_ = .047 (Nagelkerke, 1991). However, whilst the Pearson Goodness-of-Fit statistic indicated that the model was a good fit for the data (p = .352), the Deviance Goodness-of-Fit statistic indicated that overdispersion was present in the data (p = .034). Therefore, the model was re-computed using the deviance statistic’s dispersion parameter to rescale the standard errors; the results were identical. However, the effect of unusual experiences became marginal after controlling for age, gender and accuracy in the novel stimuli condition, χ^2^(2) = 5.001, p = .082. Given the marginality of this effect, the relationship between unusual experiences and accuracy was not examined further in this model.

Specifically, higher MUSEQ scores were associated with significantly greater odds of an accuracy of 50%, compared to both 0%, b = .015, Wald χ^2^(1) = 9.326, p = .003, OR = 1.015, BCa 95% CI [.005, .026], and 100%, b = .014, Wald χ^2^(1) = 4.431, p = .028, OR = 1.014, BCa 95% CI [.001, .028]. However, MUSEQ scores did not significantly predict the odds of scoring 100%, compared to 0% (p > .1).

**Table 12**

Multinomial Regression Models Examining the Effect of Depressive Symptomology, Schizotypy and Hallucination Proneness on Accuracy in the Visual Blocked Pre-Exposure Condition

| **Model** | **R^2^_N_** | **Model Statistics** | **Variable with Smallest Effect** | **Variable Statistics** |
| --- | --- | --- | --- | --- |
| **1** | .083 | χ^2^(12) = 19.232, p = .083 | Impulsive Non-Conformity | χ^2^(2) = .045, p = .978 |
| **2** | .083 | χ^2^(10) = 19.187, p = .038 | Introvertive Anhedonia | χ^2^(2) = .757, p = .685 |
| **3** | .080 | χ^2^(8) = 18.430, p = .018 | CES-D | χ^2^(2) = 1.783, p = .410 |
| **4** | .072 | χ^2^(6) = 16.647, p = .011 | Cognitive Disorganisation | χ^2^(2) = 1.386, p = .500 |
| **5** | .066 | χ^2^(4) = 15.261, p = .004 | Unusual Experiences | χ^2^(2) = 4.572, p = .102 |
| **6** | .047 | χ^2^(2) = 10.688, p = .005 | - | - |

*Note.* N = 256. The variables entered into each model are displayed below. R^2^_N_ was calculated using Nagelkerke’s (1991) equation. ‘Variable with Smallest Effect’ details the predictor with the smallest effect size; the effect selection test is reported under ‘Variable Statistics.’

1. CES-D, Unusual Experiences, Cognitive Disorganisation, Introvertive Anhedonia, Impulsive Non-Conformity and MUSEQ
2. CES-D, Unusual Experiences, Cognitive Disorganisation, Introvertive Anhedonia and MUSEQ
3. CES-D, Unusual Experiences, Cognitive Disorganisation and MUSEQ
4. Unusual Experiences, Cognitive Disorganisation and MUSEQ
5. Unusual Experiences and MUSEQ
6. MUSEQ

As unusual experiences have been reported to predict LI in previous research, the effect of unusual experiences on accuracy in the visual blocked pre-exposure condition was examined using forced entry multinomial regression. Unusual experiences significantly predicted accuracy scores, χ^2^(2) = 7.02, p = .03, R^2^_N_ = .031 (Nagelkerke, 1991). Specifically, higher unusual experiences were associated with significantly greater odds of an accuracy of 50%, compared to 100%, b = .082, Wald χ^2^(1) = 6.271, p = .012, OR = 1.085, BCa 95% CI [.019, .166]. However, unusual experiences did not significantly predict the odds of scoring 0%, compared to either 50% or 100% (p > .1).

MUSEQ and unusual experiences scores represent theoretically similar constructs and may account for similar variance in accuracy scores, resulting in a redundancy in including both variables in the model. Importantly, the effect of MUSEQ scores was similar after controlling for both unusual experiences and cognitive disorganisation, variables that affected baseline performance, suggesting MUSEQ scores specifically affect accuracy in the blocked pre-exposure condition.

In summary, these results indicate that participants with higher MUSEQ scores are more likely to get a score of 50% than either 0% or 100% in the visual blocked pre-exposure condition.

*Additional Analysis 7 – Individual Differences and Visual Mixed Pre-Exposure (Accuracy):*

Multinomial logistic regression using backwards deletion examined the effects of schizotypy, HP and DS on accuracy in the visual mixed pre-exposure condition. Exclusion of outliers resulted in 258 participants. **Table 13** details the iterations of the regression models. Across all models, multicollinearity was not considered to be problematic, as all tolerance statistics were above .2 (Menard, 2002). These results suggest that schizotypy, HP and DS did not predict accuracy in the visual mixed pre-exposure condition. Crucially, controlling for age, gender and accuracy in either the novel stimuli or blocked pre-exposure condition produced similar results.

**Table 13**

Multinomial Regression Models Examining the Effect of Depressive Symptomology, Schizotypy and Hallucination Proneness on Accuracy in the Visual Mixed Pre-Exposure Condition

| **Model** | **R^2^_N_** | **Model Statistics** | **Variable with Smallest Effect** | **Variable Statistics** |
| --- | --- | --- | --- | --- |
| **1** | .024 | χ^2^(12) = 5.473, p = .940 | Introvertive Anhedonia | χ^2^(2) = .054, p = .973 |
| **2** | .024 | χ^2^(10) = 5.419, p = .861 | Unusual Experiences | χ^2^(2) = .354, p = .838 |
| **3** | .022 | χ^2^(8) = 5.065, p = .751 | MUSEQ | χ^2^(2) = .393, p = .821 |
| **4** | .020 | χ^2^(6) = 4.672, p = .587 | CES-D | χ^2^(2) = .635, p = .728 |
| **5** | .018 | χ^2^(4) = 4.037, p = .401 | Cognitive Disorganisation | χ^2^(2) = 2.441, p = .295 |
| **6** | .007 | χ^2^(2) = 1.595, p = .450 | - | - |

*Note.* N = 258. The variables entered into each model are displayed below. R^2^_N_ was calculated using Nagelkerke’s (1991) equation. ‘Variable with Smallest Effect’ details the predictor with the smallest effect size; the effect selection test is reported under ‘Variable Statistics.’

1. CES-D, Unusual Experiences, Cognitive Disorganisation, Introvertive Anhedonia, Impulsive Non-Conformity and MUSEQ
2. CES-D, Unusual Experiences, Cognitive Disorganisation, Impulsive Non-Conformity and MUSEQ
3. CES-D, Cognitive Disorganisation, Impulsive Non-Conformity and MUSEQ
4. CES-D, Cognitive Disorganisation and Impulsive Non-Conformity
5. Cognitive Disorganisation and Impulsive Non-Conformity
6. Impulsive Non-Conformity

*Additional Analysis 8 – Individual Differences and* *Visual Mixed Pre-Exposed Common Elements with Novel Unique Elements (Reaction Times)*

Multiple regression using backwards deletion examined the effects of schizotypy, HP and DS on mean reaction time in the visual mixed pre-exposed common elements with novel unique elements condition. Exclusion of outliers resulted in 255 participants. **Table 14** details the iterations of the regression models. Across all models, the assumption of homoscedasticity was met, as indicated by inspection of plots of the predicted values against the residual values, and multicollinearity was not considered to be problematic, as all tolerance statistics were above .2 (Menard, 2002). For all analyses, the Durbin-Watson (DW) test is reported, each of which indicated that the residuals were independent. The final model indicated that reaction times were marginally predicted by MUSEQ scores, β = -.11, p = .098, BCa 95% CI [-4.523, .374]. However, this effect was abolished (p = .17) after controlling for age, gender and reaction times in the novel stimuli condition.

**Table 14**

Multiple Regression Models Examining the Effect of Depressive Symptomology, Schizotypy and Hallucination Proneness on Reaction Times in the Visual Mixed Pre-Exposed Common Elements with Novel Unique Elements Condition

| **Model** | **R** | **R^2^** | **Adjusted R^2^** | **DW** | **ANOVA Statistics** | **Variable with Smallest Effect** | **Standardised β Coefficient** | **Variable Significance** |
| --- | --- | --- | --- | --- | --- | --- | --- | --- |
| **1** | .163 | .027 | .003 | 2.152 | F(6, 248) = 1.125,  p = .348 | Introvertive Anhedonia | .012 | .851 |
| **2** | .162 | .026 | .007 | 2.150 | F(5, 249) = 1.349,  p = .244 | Cognitive Disorganisation | -.025 | .791 |
| **3** | .162 | .026 | .011 | 2.153 | F(4, 250) = 1.674,  p = .156 | CES-D | .040 | .597 |
| **4** | .158 | .025 | .013 | 2.161 | F(3, 251) = 2.145,  p = .095 | Unusual Experiences | -.073 | .417 |
| **5** | .150 | .022 | .015 | 2.158 | F(2, 252) = 2.892,  p = .057 | Impulsive Non-Conformity | .112 | .105 |
| **6** | .110 | .012 | .008 | 2.161 | F(1, 253) = 3.111,  p = .079 | - | - | - |

*Note.* N = 255. The variables entered into each model are displayed below. ‘Variable with Smallest Effect’ details the predictor with the smallest effect size; the standardised beta coefficient and associated p value of this variable are listed under ‘Standardised β Coefficient’ and ‘Variable Significance,’ respectively.

1. CES-D, Unusual Experiences, Cognitive Disorganisation, Introvertive Anhedonia, Impulsive Non-Conformity and MUSEQ
2. CES-D, Unusual Experiences, Cognitive Disorganisation, Impulsive Non-Conformity and MUSEQ
3. CES-D, Unusual Experiences, Impulsive Non-Conformity and MUSEQ
4. Unusual Experiences, Impulsive Non-Conformity and MUSEQ
5. Impulsive Non-Conformity and MUSEQ
6. MUSEQ

As unusual experiences have previously been reported to predict LI, the effect of unusual experiences in the blocked pre-exposed common elements with novel unique elements condition was examined using forced entry multiple regression. Unusual experiences failed to significantly predict reaction times, R = .086, R^2^ = .007, Adjusted R^2^ = .003, p = .173, DW = 2.178.

In summary, these results indicate that schizotypy, HP and DS did not significantly predict accuracy in the visual mixed pre-exposed common elements with novel unique elements condition, after controlling for age, gender and baseline performance.

*Additional Analysis 9 – Individual Differences and Visual Mixed Pre-Exposed Common Elements with Novel Unique Elements (Accuracy)*

Multinomial logistic regression using backwards deletion examined the effects of schizotypy, HP and DS on accuracy in the visual mixed pre-exposed common elements with novel unique elements condition. Exclusion of outliers resulted in 255 participants. **Table 15** details the iterations of the regression models. Across all models, multicollinearity was not considered to be problematic, as all tolerance statistics were above .2 (Menard, 2002). Also, the assumption of linearity was met across all models, as indicated by the non-significant (p > .05) contribution of the interaction between predictors and the log of the respective predictors to each model (Field, 2013).

The final model indicated that both unusual experiences, χ^2^(2) = 12.207, p = .002, and MUSEQ scores, χ^2^(2) = 6.565, p = .038, significantly predicted accuracy, χ^2^(4) = 12.356, p = .015, R^2^_N_ = .054 (Nagelkerke, 1991). Importantly, the Pearson and Deviance Goodness-of-Fit statistics both indicated that model fit was adequate, as the model’s predicted and observed values did not significantly differ (p > .05). Crucially, controlling for age, gender and reaction times in the novel stimuli condition produced similar results.

Specifically, higher unusual experiences were associated with significantly lower odds of an accuracy of 50%, compared to 0%, b = -.115, Wald χ^2^(1) = 11.217, p = .001, OR = .891, BCa 95% CI [-.188, -.052], and marginally lower odds of an accuracy of 50%, compared to 100%, b = -.071, Wald χ^2^(1) = 3.344, p = .072, OR = .931, BCa 95% CI [-.154, .003]. Also, higher MUSEQ scores were associated with significantly greater odds of an accuracy of 50%, compared to 0%, b = -.018, Wald χ^2^(1) = 6.289, p = .013, OR = 1.018, BCa 95% CI [.003, .035]. However, MUSEQ scores did not predict the odds of scoring 100%, compared to either 0% or 50% (p > .1). Importantly, the effect of unusual experiences and MUSEQ scores was similar after controlling for cognitive disorganisation, a variable that affected baseline performance.

**Table 15**

Multinomial Regression Models Examining the Effect of Depressive Symptomology, Schizotypy and Hallucination Proneness on Accuracy in the Visual Mixed Pre-Exposed Common Elements with Novel Unique Elements Condition

| **Model** | **R^2^_N_** | **Model Statistics** | **Variable with Smallest Effect** | **Variable Statistics** |
| --- | --- | --- | --- | --- |
| **1** | .069 | χ^2^(12) = 16.145, p = .185 | Cognitive Disorganisation | χ^2^(2) = .284, p = .868 |
| **2** | .068 | χ^2^(10) = 15.861, p = .104 | Impulsive Non-Conformity | χ^2^(2) = .368, p = .832 |
| **3** | .067 | χ^2^(8) = 15.493, p = .050 | Introvertive Anhedonia | χ^2^(2) = 1.230, p = .541 |
| **4** | .062 | χ^2^(6) = 14.263, p = .027 | CES-D | χ^2^(2) = 1.907, p = .385 |
| **5** | .054 | χ^2^(4) = 12.356, p = .015 | - | - |

*Note.* N = 255. The variables entered into each model are displayed below. R^2^_N_ was calculated using Nagelkerke’s (1991) equation. ‘Variable with Smallest Effect’ details the predictor with the smallest effect size; the effect selection test is reported under ‘Variable Statistics.’

1. CES-D, Unusual Experiences, Cognitive Disorganisation, Introvertive Anhedonia, Impulsive Non-Conformity and MUSEQ
2. CES-D, Unusual Experiences, Introvertive Anhedonia, Impulsive Non-Conformity and MUSEQ
3. CES-D, Unusual Experiences, Introvertive Anhedonia and MUSEQ
4. CES-D, Unusual Experiences and MUSEQ
5. Unusual Experiences and MUSEQ

In summary, these results indicate that participants with greater unusual experiences are more likely to get a score of 0% than 50% and are marginally more likely to get a score of 100% than 50%. Furthermore, they indicate that participants with greater MUSEQ scores are more likely to get a score of 50% than 0%.

*Additional Analysis 10 – Individual Differences and* *Visual Blocked Pre-Exposed Common Elements with Novel Unique Elements (Reaction Times)*

Multiple regression using backwards deletion examined the effects of schizotypy, HP and DS on mean reaction time in the visual blocked pre-exposed common elements with novel unique elements condition. Exclusion of outliers resulted in 255 participants. **Table 16** details the iterations of the regression models. Across all models, the assumption of homoscedasticity was met, as indicated by inspection of plots of the predicted values against the residual values, and multicollinearity was not considered to be problematic, as all tolerance statistics were above .2 (Menard, 2002). For all analyses, the Durbin-Watson (DW) test is reported, each of which indicated that the residuals were independent. These results suggest that schizotypy, HP and DS did not predict reaction times in the visual blocked pre-exposed common elements with novel unique elements condition.

**Table 16**

Multiple Regression Models Examining the Effect of Depressive Symptomology, Schizotypy and Hallucination Proneness on Reaction Times in the Visual Blocked Pre-Exposed Common Elements with Novel Unique Elements Condition

| **Model** | **R** | **R^2^** | **Adjusted R^2^** | **DW** | **ANOVA Statistics** | **Variable with Smallest Effect** | **Standardised β Coefficient** | **Variable Significance** |
| --- | --- | --- | --- | --- | --- | --- | --- | --- |
| **1** | .194 | .038 | .014 | 1.916 | F(6, 248) = 1.621,  p = .142 | Cognitive Disorganisation | -.038 | .688 |
| **2** | .193 | .037 | .018 | 1.912 | F(5, 249) = 1.919,  p = .092 | Unusual Experiences | .050 | .590 |
| **3** | .190 | .036 | .021 | 1.902 | F(4, 250) = 2.333,  p = .056 | CES-D | .078 | .283 |
| **4** | .178 | .032 | .020 | 1.909 | F(3, 251) = 2.723,  p = .045 | Introvertive Anhedonia | -.079 | .106 |
| **5** | .159 | .025 | .018 | 1.902 | F(2, 252) = 3.272,  p = .040 | Impulsive Non-Conformity | .133 | .052 |
| **6** | .103 | .011 | .007 | 1.870 | F(1, 253) = 2.715,  p = .101 | - | - | - |

*Note.* N = 255. The variables entered into each model are displayed below. ‘Variable with Smallest Effect’ details the predictor with the smallest effect size; the standardised beta coefficient and associated p value of this variable are listed under ‘Standardised β Coefficient’ and ‘Variable Significance,’ respectively.

1. CES-D, Unusual Experiences, Cognitive Disorganisation, Introvertive Anhedonia, Impulsive Non-Conformity and MUSEQ
2. CES-D, Unusual Experiences, Introvertive Anhedonia, Impulsive Non-Conformity and MUSEQ
3. CES-D, Introvertive Anhedonia, Impulsive Non-Conformity and MUSEQ
4. Introvertive Anhedonia, Impulsive Non-Conformity and MUSEQ
5. Impulsive Non-Conformity and MUSEQ
6. MUSEQ

As unusual experiences have previously been reported to predict LI, the effect of unusual experiences in the blocked pre-exposed common elements with novel unique elements condition was examined using forced entry multiple regression. Unusual experiences failed to significantly predict reaction times, R = .011, R^2^ < .001, Adjusted R^2^ = -.004, p = .863, DW = 1.902.

*Additional Analysis 11 – Individual Differences and* *Visual Blocked Pre-Exposed Common Elements with Novel Unique Elements (Accuracy)*

Multinomial logistic regression using backwards deletion examined the effects of schizotypy, HP and DS on accuracy in the visual blocked pre-exposed common elements with novel unique elements condition. Exclusion of outliers resulted in 255 participants. **Table 17** details the iterations of the regression models. Across all models, multicollinearity was not considered to be problematic, as all tolerance statistics were above .2 (Menard, 2002). The final model indicated that CES-D scores marginally predicted accuracy, χ^2^(2) = 5.537, p = .063, R^2^_N_ = .024 (Nagelkerke, 1991). Importantly, the Pearson and Deviance Goodness-of-Fit statistics both indicated that model fit was adequate, as the model’s predicted and observed values did not significantly differ (p > .05). Crucially, controlling for age, gender and accuracy in the novel stimuli condition produced similar results. Given the marginality of these effects, these relationships were not examined further.

**Table 17**

Multinomial Regression Models Examining the Effect of Depressive Symptomology, Schizotypy and Hallucination Proneness on Accuracy in the Visual Blocked Pre-Exposed Common Elements with Novel Unique Elements Condition

| **Model** | **R^2^_N_** | **Model Statistics** | **Variable with Smallest Effect** | **Variable Statistics** |
| --- | --- | --- | --- | --- |
| **1** | .065 | χ^2^(12) = 15.103, p = .236 | Cognitive Disorganisation | χ^2^(2) = .517, p = .772 |
| **2** | .063 | χ^2^(10) = 14.586, p = .148 | MUSEQ | χ^2^(2) = 1.942, p = .379 |
| **3** | .055 | χ^2^(8) = 12.644, p = .125 | Introvertive Anhedonia | χ^2^(2) = 1.992, p = .369 |
| **4** | .046 | χ^2^(6) = 10.652, p = .100 | Unusual Experiences | χ^2^(2) = 2.939, p = .230 |
| **5** | .034 | χ^2^(4) = 7.713, p = .103 | Impulsive Non-Conformity | χ^2^(2) = 2.175, p = .337 |
| **6** | .024 | χ^2^(2) = 5.537, p = .063 | - | - |

*Note.* N = 255. The variables entered into each model are displayed below. R^2^_N_ was calculated using Nagelkerke’s (1991) equation. ‘Variable with Smallest Effect’ details the predictor with the smallest effect size; the effect selection test is reported under ‘Variable Statistics.’

1. CES-D, Unusual Experiences, Cognitive Disorganisation, Introvertive Anhedonia, Impulsive Non-Conformity and MUSEQ
2. CES-D, Unusual Experiences, Introvertive Anhedonia, Impulsive Non-Conformity and MUSEQ
3. CES-D, Unusual Experiences, Introvertive Anhedonia and Impulsive Non-Conformity
4. CES-D, Unusual Experiences and Impulsive Non-Conformity
5. CES-D and Impulsive Non-Conformity
6. CES-D

As unusual experiences scores have previously been reported to predict LI, the effect of unusual experiences on accuracy was examined using forced entry multinomial regression. Unusual experiences did not significantly affect accuracy, χ^2^(2) = 3.958, p = .138, R^2^_N_ = .017 (Nagelkerke, 1991).

In summary, these results indicate that schizotypy, HP and DS did not statistically significantly (p < .05) predict accuracy in the visual blocked pre-exposed common elements with novel unique elements condition.

*Additional Analysis 12 – Individual Differences and Visual Mixed Pre-Exposed Unique Elements with Novel Common Unique Elements (Reaction Times)*

Multiple regression using backwards deletion examined the effects of schizotypy, HP and DS on mean reaction time in the visual mixed pre-exposed unique elements with novel common elements condition. Exclusion of outliers resulted in 256 participants. **Table 18** details the iterations of the regression models. Across all models, the assumption of homoscedasticity was met, as indicated by inspection of plots of the predicted values against the residual values, and multicollinearity was not considered to be problematic, as all tolerance statistics were above .2 (Menard, 2002). For all analyses, the Durbin-Watson (DW) test is reported, each of which indicated that the residuals were independent. The final model marginally predicted reaction times, F(2, 253) = 2.905, p = .057. Specifically, reaction times were significantly predicted by MUSEQ scores, β = -.140, p = .02, BCa 95% CI [-4.538, -.523], and marginally predicted by impulsive non-conformity, β = .139, p = .068, BCa 95% CI [2.956, 52.521]. However, controlling for age, gender and reaction times in the novel stimuli condition resulted in both MUSEQ scores, β = -.111 p = .075, BCa 95% CI [-4.495, -.010], and impulsive non-conformity, β = .132, p = .089, BCa 95% CI [-.011, 56.444], maintaining only marginal effects.

**Table 18**

Multiple Regression Models Examining the Effect of Depressive Symptomology, Schizotypy and Hallucination Proneness on Reaction Times in the Visual Mixed Pre-Exposed Unique Elements with Novel Common Unique Elements Condition

| **Model** | **R** | **R^2^** | **Adjusted R^2^** | **DW** | **ANOVA Statistics** | **Variable with Smallest Effect** | **Standardised β Coefficient** | **Variable Significance** |
| --- | --- | --- | --- | --- | --- | --- | --- | --- |
| **1** | .207 | .043 | .020 | 2.111 | F(6, 249) = 1.861,  p = .088 | Cognitive Disorganisation | -.083 | .382 |
| **2** | .200 | .040 | .021 | 2.102 | F(5, 250) = 2.081,  p = .068 | Introvertive Anhedonia | -.066 | .304 |
| **3** | .189 | .036 | .021 | 2.117 | F(4, 251) = 2.335,  p = .056 | Unusual Experiences | -.123 | .192 |
| **4** | .171 | .029 | .018 | 2.115 | F(3, 252) = 2.535,  p = .057 | CES-D | .095 | .183 |
| **5** | .150 | .022 | .015 | 2.120 | F(2, 253) = 2.905,  p = .057 | - | - | - |

*Note.* N = 256. The variables entered into each model are displayed below. ‘Variable with Smallest Effect’ details the predictor with the smallest effect size; the standardised beta coefficient and associated p value of this variable are listed under ‘Standardised β Coefficient’ and ‘Variable Significance,’ respectively.

1. CES-D, Unusual Experiences, Cognitive Disorganisation, Introvertive Anhedonia, Impulsive Non-Conformity and MUSEQ
2. CES-D, Unusual Experiences, Introvertive Anhedonia, Impulsive Non-Conformity and MUSEQ
3. CES-D, Unusual Experiences, Impulsive Non-Conformity and MUSEQ
4. CES-D, Impulsive Non-Conformity and MUSEQ
5. Impulsive Non-Conformity and MUSEQ

In summary, these results indicate that lower MUSEQ scores and higher impulsive non-conformity scores may predict slower reaction times in the mixed pre-exposed unique elements with novel common elements condition. However, caution is necessary, given the marginality of these effects and the unreliability of the impulsive non-conformity dimension.

*Additional Analysis 13 – Individual Differences and* *Visual Mixed Pre-Exposed Unique Elements with Novel Common Unique Elements (Accuracy)*

Multinomial logistic regression using backwards deletion examined the effects of schizotypy, HP and DS on accuracy in the visual mixed pre-exposed unique elements with novel common elements condition. Exclusion of outliers resulted in 256 participants. **Table 19** details the iterations of the regression models. Across all models, multicollinearity was not considered to be problematic, as all tolerance statistics were above .2 (Menard, 2002). These results indicate that schizotypy, HP and DS did not significantly predict accuracy in the visual mixed pre-exposed unique elements with novel common elements condition.

**Table 19**

Multinomial Regression Models Examining the Effect of Depressive Symptomology, Schizotypy and Hallucination Proneness on Accuracy in the Visual Mixed Pre-Exposed Unique Elements with Novel Common Unique Elements Condition

| **Model** | **R^2^_N_** | **Model Statistics** | **Variable with Smallest Effect** | **Variable Statistics** |
| --- | --- | --- | --- | --- |
| **1** | .051 | χ^2^(12) = 11.872, p = .456 | Introvertive Anhedonia | χ^2^(2) = .434, p = .805 |
| **2** | .049 | χ^2^(10) = 11.438, p = .324 | MUSEQ | χ^2^(2) = .656, p = .720 |
| **3** | .046 | χ^2^(8) = 10.783, p = .214 | Unusual Experiences | χ^2^(2) = 1.402, p = .496 |
| **4** | .041 | χ^2^(6) = 9.381, p = .153 | Impulsive Non-Conformity | χ^2^(2) = 3.264, p = .196 |
| **5** | .027 | χ^2^(4) = 6.117, p = .191 | CES-D | χ^2^(2) = 3.391, p = .183 |
| **6** | .012 | χ^2^(2) = 2.726, p = .256 | - | - |

*Note.* N = 256. The variables entered into each model are displayed below. R^2^_N_ was calculated using Nagelkerke’s (1991) equation. ‘Variable with Smallest Effect’ details the predictor with the smallest effect size; the effect selection test is reported under ‘Variable Statistics.’

1. CES-D, Unusual Experiences, Cognitive Disorganisation, Introvertive Anhedonia, Impulsive Non-Conformity and MUSEQ
2. CES-D, Unusual Experiences, Cognitive Disorganisation, Impulsive Non-Conformity and MUSEQ
3. CES-D, Unusual Experiences, Cognitive Disorganisation and Impulsive Non-Conformity
4. CES-D, Cognitive Disorganisation and Impulsive Non-Conformity
5. CES-D and Cognitive Disorganisation
6. Cognitive Disorganisation

*Additional Analysis 14 – Individual Differences and Visual Blocked Pre-Exposed Unique Elements with Novel Common Elements (Reaction Times)*

Multiple regression using backwards deletion examined the effects of schizotypy, HP and DS on mean reaction time in the visual blocked pre-exposed unique elements with novel common elements condition. Exclusion of outliers resulted in 258 participants. **Table 20** details the iterations of the regression models. Across all models, the assumption of homoscedasticity was met, as indicated by inspection of plots of the predicted values against the residual values, and multicollinearity was not considered to be problematic, as all tolerance statistics were above .2 (Menard, 2002). For all analyses, the Durbin-Watson (DW) test is reported, each of which indicated that the residuals were independent. These results indicate that schizotypy, HP and DS did not significantly predict reaction times in the visual blocked pre-exposed unique elements with novel common elements condition.

**Table 20**

Multiple Regression Models Examining the Effect of Depressive Symptomology, Schizotypy and Hallucination Proneness on Reaction Times in the Visual Blocked Pre-Exposed Unique Elements with Novel Common Elements Condition

| **Model** | **R** | **R^2^** | **Adjusted R^2^** | **DW** | **ANOVA Statistics** | **Variable with Smallest Effect** | **Standardised β Coefficient** | **Variable Significance** |
| --- | --- | --- | --- | --- | --- | --- | --- | --- |
| **1** | .102 | .010 | -.013 | 2.137 | F(6, 251) = .442,  p = .850 | Cognitive Disorganisation | -.005 | .959 |
| **2** | .102 | .010 | -.009 | 2.136 | F(5, 252) = .532,  p = .752 | Unusual Experiences | -.010 | .919 |
| **3** | .102 | .010 | -.005 | 2.137 | F(4, 253) = .665,  p = .617 | Impulsive Non-Conformity | -.023 | .748 |
| **4** | .100 | .010 | -.002 | 2.137 | F(3, 254) = .855,  p = .465 | MUSEQ | .031 | .662 |
| **5** | .096 | .009 | .001 | 2.133 | F(2, 255) = 1.191,  p = .306 | CES-D | -.073 | .257 |
| **6** | .065 | .004 | < .001 | 2.132 | F(1, 256) = 1.089,  p = .298 | - | - | - |

*Note.* N = 258. The variables entered into each model are displayed below. ‘Variable with Smallest Effect’ details the predictor with the smallest effect size; the standardised beta coefficient and associated p value of this variable are listed under ‘Standardised β Coefficient’ and ‘Variable Significance,’ respectively.

1. CES-D, Unusual Experiences, Cognitive Disorganisation, Introvertive Anhedonia, Impulsive Non-Conformity and MUSEQ
2. CES-D, Unusual Experiences, Introvertive Anhedonia, Impulsive Non-Conformity and MUSEQ
3. CES-D, Introvertive Anhedonia, Impulsive Non-Conformity and MUSEQ
4. CES-D, Introvertive Anhedonia and MUSEQ
5. CES-D and Introvertive Anhedonia
6. Introvertive Anhedonia

*Additional Analysis 15 – Individual Differences and Visual Blocked Pre-Exposed Unique Elements with Novel Common Unique Elements (Accuracy)*

Multinomial logistic regression using backwards deletion examined the effects of schizotypy, HP and DS on accuracy in the visual blocked pre-exposed unique elements with novel common elements condition. Exclusion of outliers resulted in 258 participants. **Table 21** details the iterations of the regression models. Across all models, multicollinearity was not considered to be problematic, as all tolerance statistics were above .2 (Menard, 2002). The final model indicated that cognitive disorganisation significantly predicted accuracy, χ^2^(2) = 6.441, p = .04, R^2^_N_ = .028 (Nagelkerke, 1991). Importantly, the Pearson and Deviance Goodness-of-Fit statistics both indicated that model fit was adequate, as the model’s predicted and observed values did not significantly differ (p > .05). However, the model was no longer statistically significant after controlling for age, gender and accuracy in the novel stimuli condition (p > .1).

**Table 21**

Multinomial Regression Models Examining the Effect of Depressive Symptomology, Schizotypy and Hallucination Proneness on Accuracy in the Visual Blocked Pre-Exposed Unique Elements with Novel Common Unique Elements Condition

| **Model** | **R^2^_N_** | **Model Statistics** | **Variable with Smallest Effect** | **Variable Statistics** |
| --- | --- | --- | --- | --- |
| **1** | .045 | χ^2^(12) = 10.403, p = .581 | CES-D | χ^2^(2) = .617, p = .735 |
| **2** | .042 | χ^2^(10) = 9.786, p = .459 | Introvertive Anhedonia | χ^2^(2) = .608, p = .738 |
| **3** | .039 | χ^2^(8) = 9.178, p = .327 | MUSEQ | χ^2^(2) = .845, p = .655 |
| **4** | .036 | χ^2^(6) = 8.333, p = .215 | Unusual Experiences | χ^2^(2) = .484, p = .785 |
| **5** | .034 | χ^2^(4) = 7.849, p = .097 | Impulsive Non-Conformity | χ^2^(2) = 1.408, p = .495 |
| **6** | .028 | χ^2^(2) = 6.441, p = .040 | - | - |

*Note.* N = 258. The variables entered into each model are displayed below. R^2^_N_ was calculated using Nagelkerke’s (1991) equation. ‘Variable with Smallest Effect’ details the predictor with the smallest effect size; the effect selection test is reported under ‘Variable Statistics.’

1. CES-D, Unusual Experiences, Cognitive Disorganisation, Introvertive Anhedonia, Impulsive Non-Conformity and MUSEQ
2. Unusual Experiences, Cognitive Disorganisation, Introvertive Anhedonia, Impulsive Non-Conformity and MUSEQ
3. Unusual Experiences, Cognitive Disorganisation, Impulsive Non-Conformity and MUSEQ
4. Unusual Experiences, Cognitive Disorganisation and Impulsive Non-Conformity
5. Cognitive Disorganisation and Impulsive Non-Conformity
6. Cognitive Disorganisation

In summary, these results suggest that schizotypy, HP and DS did not statistically significantly (p < .05) predict accuracy in the visual blocked pre-exposed unique elements with novel common elements condition.

*Additional Analysis 16 – Visual Perceptual Learning for the Same Stimuli (Reaction Times):*

A one-way repeated measures ANOVA examined whether reaction times in responding to the same visual stimuli varied between the mixed pre-exposure, blocked pre-exposure and novel stimuli conditions. Exclusion of outliers resulted in 255 participants. The assumption of sphericity was met for the main effect of pre-exposure condition, χ^2^(2) = 4.803, p = .091.

After these provisional analyses, the main effect was examined. **Table** **22** details the means and standard deviations of participants’ reaction times in each condition. The results indicate that the main effect of pre-exposure condition significantly affected reaction times, F(2, 508) = 4.732, p = .009, η_p_^2^ = .018; this represents a small effect size (Lakens, 2013). Post-hoc pairwise comparisons using the Bonferroni correction indicated that participants exhibited significantly faster reaction times in the mixed pre-exposure (M = 918, SD = 560), compared to the novel stimuli (M = 1039, SD = 540), condition (p = .017) and in the blocked pre-exposure (M = 930, SD = 521), compared to the novel stimuli, condition (p = .023). However, reaction times did not significantly differ between the mixed and blocked pre-exposure conditions (p = 1).

**Table 22**

Means and Standard Deviations of Reaction Times to the Same Stimuli in the Visual Mixed Pre-Exposure, Blocked Pre-Exposure and Novel Stimuli Conditions

| **Pre-Exposure Condition** | **Mean** | **Standard Deviation** |
| --- | --- | --- |
| **Mixed Pre-Exposure** | 918 | 560 |
| **Blocked Pre-Exposure** | 930 | 521 |
| **Novel Stimuli** | 1039 | 540 |

*Note.* N = 255. Reaction times are reported in milliseconds.

*Additional Analysis 17 – Visual Pre-Exposed Common Elements with Novel Unique Elements for the Same Stimuli (Reaction Times)*

A one-way repeated measures ANOVA examined whether reaction times in responding to the same visual stimuli varied between the mixed pre-exposed common elements with novel unique elements, blocked pre-exposed common elements with novel unique elements and novel stimuli conditions. Seven outliers were removed, resulting in 253 cases. The assumption of sphericity was met for the main effect of pre-exposure condition, χ^2^(2) = 1.556, p = .459.

After these provisional analyses, the main effect was examined. **Table 23** details the means and standard deviations of participants’ reaction times. The results indicate that reaction times significantly differed between pre-exposure conditions, F(2, 504) = 5.683, p = .004, η_p_^2^ = .022; this represents a small effect size (Lakens, 2013). Post-hoc pairwise comparisons using the Bonferroni correction indicated that participants exhibited significantly faster reaction times in the mixed pre-exposed common elements with novel unique elements (M = 928, SD = 529), compared to the novel stimuli (M = 1032, SD = 521), condition (p = .032) and in the blocked pre-exposed common elements with novel unique elements (M = 909, SD = 452), compared to the novel stimuli, condition (p = .004). However, reaction times did not significantly differ between the mixed and blocked pre-exposed common elements with novel unique elements conditions (p = 1).

**Table 23**

Means and Standard Deviations of Reaction Times to the Same Stimuli in the Visual Mixed Pre-Exposed Common Elements with Novel Unique Elements, Blocked Pre-Exposed Common Elements with Novel Unique Elements and Novel Stimuli Conditions

| **Pre-Exposure Condition** | **Mean** | **Standard Deviation** |
| --- | --- | --- |
| **Mixed Pre-Exposed Common and Novel Unique Elements** | 928 | 529 |
| **Blocked Pre-Exposed Common and Novel Unique Elements** | 909 | 452 |
| **Novel Stimuli** | 1032 | 521 |

*Note.* N = 253. Reaction times are reported in milliseconds.

*Additional Analysis 18 – Visual Pre-Exposed Unique Elements with Novel Common Elements for the Same Stimuli (Reaction Time):*

A one-way repeated measures ANOVA examined whether reaction times in responding to the same visual stimuli varied between the mixed pre-exposed unique elements with novel common elements, blocked pre-exposed unique elements with novel common elements and novel stimuli conditions. Six outliers were removed, resulting in 254 cases. The assumption of sphericity was met for the main effect of pre-exposure condition, χ^2^(2) = 3.772, p = .152.

After these provisional analyses, the main effect was examined. **Table 24** details the means and standard deviations of participants’ reaction times. The results indicate that reaction times significantly differed with respect to pre-exposure condition, F(2, 506) = 8.625, p < .001, η_p_^2^ = .033; this represents a small effect size (Lakens, 2013). Post-hoc pairwise comparisons using the Bonferroni correction indicated that participants exhibited significantly faster reaction times in the mixed pre-exposed unique elements with novel common elements (M = 899, SD = 464), compared to the novel stimuli (M = 1044, SD = 544), condition (p = .001) and in the blocked pre-exposed unique elements with novel common elements (M = 916, SD = 460), compared to the novel stimuli, condition (p = .004). However, reaction times did not significantly differ between the mixed and blocked pre-exposed unique elements with novel common elements conditions (p = 1).

**Table 24**

Means and Standard Deviations of Reaction Times to the Same Stimuli in the Visual Mixed Pre-Exposed Unique Elements with Novel Common Elements, Blocked Pre-Exposed Unique Elements with Novel Common Elements and Novel Stimuli Conditions

| **Pre-Exposure Condition** | **Mean** | **Standard Deviation** |
| --- | --- | --- |
| **Mixed Pre-Exposed Unique and Novel Common Elements** | 899 | 464 |
| **Blocked Pre-Exposed Unique and Novel Common Elements** | 916 | 460 |
| **Novel Stimuli** | 1044 | 544 |

*Note.* N = 254. Reaction times are reported in milliseconds.

*Additional Analysis 19 – Individual Differences and Baseline Visual Perceptual Discrimination for the Same Stimuli (Accuracy):*

Forced entry multinomial logistic regression examined the effect of unusual experiences and cognitive disorganisation on accuracy in discriminating between the same novel visual. Exclusion of outliers resulted in 257 participants. Multicollinearity was not considered to be problematic, as all tolerance statistics were above .2 (Menard, 2002). The results suggested that unusual experiences and cognitive disorganisation did not predict accuracy in discriminating between the same novel visual stimuli, χ^2^(4) = 5.923, p = .205.

*Additional Analysis 20 – Individual Differences and Visual Blocked Pre-Exposure for the Same Stimuli (Accuracy):*

Forced entry multinomial logistic regression examined the effect of MUSEQ scores on accuracy in discriminating between the same visual stimuli after blocked pre-exposure. Exclusion of outliers resulted in 256 participants. The results suggested that MUSEQ scores did not predict accuracy in discriminating between the same visual stimuli after blocked pre-exposure, χ^2^(2) = .067, p = .967. In a second model, forced entry multinomial logistic regression examined the effect of unusual experiences scores on accuracy in discriminating between the same visual stimuli after blocked pre-exposure. The results suggested that unusual experiences scores did not predict accuracy in discriminating between the same visual stimuli after blocked pre-exposure, χ^2^(2) = .912, p = .634.

*Additional Analysis 21 – Individual Differences and Visual Mixed Pre-Exposed Common Elements with Novel Unique Elements for the Same Stimuli (Accuracy):*

Forced entry multinomial logistic regression examined the effect of unusual experiences and MUSEQ scores on accuracy in the visual mixed pre-exposed common elements with novel unique elements condition. Exclusion of outliers resulted in 254 participants. Multicollinearity was not considered to be problematic, as all tolerance statistics were above .2 (Menard, 2002). The results suggested that unusual experiences and MUSEQ scores did not predict accuracy in discriminating between the same stimuli in the visual mixed pre-exposed common elements with novel unique elements condition, χ^2^(4) = 1.767, p = .778.

*Additional Analysis 22 – Individual Differences and Visual Mixed Pre-Exposure for the Same Stimuli (Reaction Times):*

Forced entry multiple regression examined the effect of unusual experiences and cognitive disorganisation on reaction times in discriminating between the same stimuli in the visual mixed pre-exposure condition. One outlier was excluded, resulting in a total of 259 participants. The assumption of homoscedasticity was met, as indicated by inspection of a plot of the predicted values against the residual values, and multicollinearity was not considered to be problematic, as all tolerance statistics were above .2 (Menard, 2002). The Durbin-Watson (DW) test suggested that the residuals were independent (DW = 1.892). Unusual experiences and cognitive disorganisation did not predict reaction times in discriminating between the same stimuli in the visual mixed pre-exposure condition, R = .031, R^2^ = .001, Adjusted R^2^ = -.007, p = .883.

*Additional Analysis 23 – Perceptual Learning Planned Pairwise Comparisons*

Planned Wilcoxon (1945) matched-pair signed-rank tests examined whether accuracy differed between the mixed pre-exposure, blocked pre-exposure and novel stimuli conditions in the visual PL task.

In the visual PL task, participants were significantly more accurate at discriminating between similar stimuli following mixed, compared to blocked, pre-exposure, T = 4810, p = .046 (one-tailed), r = .075. However, accuracy did not significantly differ between the novel stimuli condition and either the mixed pre-exposure, T = 6324, p = .205 (one-tailed), r = .036, or blocked pre-exposure conditions, T = 6308, p = .169 (one-tailed), r = .042.

## **Appendix 7: Intercorrelations Between Scale Items and Performance**

**Table 25**

*Intercorrelations Between Unusual Experiences Scale Items and Visual Latent Inhibition (Accuracy)*

| **Unusual Experiences Scale Item** | **Latent Inhibition** |  | **Unusual Experiences Scale Item** | **Latent Inhibition** |
| --- | --- | --- | --- | --- |
| **1** | -.070 |  | **16** | -.059 |
| **2** | -.107 |  | **17** | -.059 |
| **3** | -.055 |  | **18** | -.054 |
| **4** | -.004 |  | **19** | < .001 |
| **5** | -.010 |  | **20** | .006 |
| **6** | -.034 |  | **21** | -.075 |
| **7** | -.007 |  | **22** | -.032 |
| **8** | -.104 |  | **23** | -.036 |
| **9** | -.014 |  | **24** | -.047 |
| **10** | .050 |  | **25** | .007 |
| **11** | -.065 |  | **26** | -.076 |
| **12** | -.108 |  | **27** | .003 |
| **13** | -.200** |  | **28** | -.024 |
| **14** | -.002 |  | **29** | -.030 |
| **15** | < .001 |  | **30** | -.083 |

*Note.* N = 260. Pearson’s product-moment correlation coefficients are shown in the table.
** p < .01.

**Table 26**

*Intercorrelations Between Unusual Experiences Scale Items and Visual Conditioned Inhibition (Reaction Times)*

| **Unusual Experiences Scale Item** | **Conditioned Inhibition** |  | **Unusual Experiences Scale Item** | **Conditioned Inhibition** |
| --- | --- | --- | --- | --- |
| **1** | -.006 |  | **16** | -.038 |
| **2** | .124* |  | **17** | -.092 |
| **3** | .073 |  | **18** | -.018 |
| **4** | -.121 |  | **19** | < .001 |
| **5** | -.007 |  | **20** | -.011 |
| **6** | -.036 |  | **21** | -.143* |
| **7** | .010 |  | **22** | .053 |
| **8** | -.140* |  | **23** | .065 |
| **9** | -.105 |  | **24** | -.058 |
| **10** | .041 |  | **25** | -.053 |
| **11** | -.003 |  | **26** | -.070 |
| **12** | .022 |  | **27** | -.010 |
| **13** | -.026 |  | **28** | -.016 |
| **14** | .001 |  | **29** | -.149* |
| **15** | -.092 |  | **30** | -.033 |

*Note.* N = 255. Pearson’s product-moment correlation coefficients are shown in the table.
* p < .05.

**Table 27**

*Intercorrelations Between Cognitive Disorganisation Scale Items and Visual Conditioned Inhibition (Reaction Times)*

| **Cognitive Disorganisation Scale Item** | **Conditioned Inhibition** |  | **Cognitive Disorganisation Scale Item** | **Conditioned Inhibition** |
| --- | --- | --- | --- | --- |
| **1** | .016 |  | **13** | .095 |
| **2** | .114 |  | **14** | .023 |
| **3** | .021 |  | **15** | .076 |
| **4** | .035 |  | **16** | .069 |
| **5** | -.073 |  | **17** | .124* |
| **6** | .004 |  | **18** | .196** |
| **7** | .065 |  | **19** | .052 |
| **8** | .105 |  | **20** | .018 |
| **9** | .078 |  | **21** | -.019 |
| **10** | .025 |  | **22** | .058 |
| **11** | -.080 |  | **23** | .064 |
| **12** | .001 |  | **24** | .083 |

*Note.* N = 259. Pearson’s product-moment correlation coefficients are shown in the table.
* p < .05. ** p < .01.

## **Appendix 8: Aberrant Salience and Depression**

In this study, the Centre for Epidemiologic Studies Depression Scale (CES-D) was included as a measure of depressive symptomology (Radloff, 1977). This aimed to evaluate a theory regarding the role of aberrant salience in depression (see Myles, 2024). Given space limitations, it has not been possible to explicate this theory in the main text. However, a quotation from Myles (2024) is provided below:

“Whilst the Salience Hypothesis provides a comprehensive account of hallucinations and unusual beliefs, Kapur et al. (2005) acknowledged that it fails to account for commonly co-occurring symptoms, including depression. Depressive symptomology (DS), characterised by low mood, despondency and loss of interest in pleasurable activities (APA, 2013), is experienced by up to 80% of people with schizophrenia (Buckley & Castle, 2015; Upthegrove, Marwaha & Birchwood, 2017; Winograd-Gurvich, Fitzgerald, Georgiou-Karistianis, Bradshaw & White, 2006) and suicide risk is up to 13 times higher than in the general population (Hayes et al., 2012), though this varies with demographic factors (Majadas, Olivares, Galan & Diez, 2012; Subodh & Grover, 2020). Furthermore, DS correlates with fluctuations in positive symptoms to a greater extent than negative symptoms in both people with schizophrenia (Lysaker, Bell, Bioty & Zito, 1995; Norman & Malla, 1994) and neurotypicals (Lewandowski et al., 2006), providing an, albeit highly tentative, indication that these symptoms may partially be underpinned by similar cognitive processes. Despite this, contemporary theories lack a parsimonious account of hallucinations, unusual beliefs and DS that appeals to a single cognitive mechanism rather than several separate comorbid deficits.

However, this thesis argues that the Salience Hypothesis can be extended to account for DS in schizophrenia. First, it is necessary to elucidate a common cause of DS. Seminal research indicates that low perceived control (PC), defined as the perceived absence of the capability to influence environmental contingencies (Seligman, 1975), maintains a causal role in depression (Abramson, Metalsky & Alloy, 1989; Abramson, Seligman & Teasdale, 1978; Alloy & Abramson, 1979; Bandura, 2010; Seligman, 1975; Seligman, 1992). Evidence for this comes from cross-sectional demonstrations that individuals with lower PC exhibit greater DS (Bjørkløf et al., 2016; Bostik & Everall, 2007; Cheng, Cheung, Chio & Chan, 2013; Crandall, Allsop & Hanson, 2018; Crona, Stenmarker, Öjehagen, Hallberg & Brådvik, 2017; Kleinberg, Aluoja & Vasar, 2013; Myles, Connolly & Stanulewicz, 2020; Nicolopoulos, Boydell, Shand & Christensen, 2018; Volz, Voelkle & Werheid, 2018). Moreover, longitudinal studies indicate that low PC predicts the subsequent manifestation of depression, suggesting PC maintains a causal role in DS (Bjørkløf et al., 2018; Galla & Wood, 2015; Hamilton & Abramson, 1983; Millman et al., 2017; Tobin & Raymundo, 2010). These robust, replicable findings provide strong evidence that low PC increases one’s vulnerability to depression.

This thesis proposes that low PC may also account for DS in schizophrenia. Perceived control can be conceptualised as an association between environmental outcomes (US) and an agent’s actions (CS; Seligman, 1975). Crucially, contextual stimuli, referring to all other stimuli that the agent has encoded, also enter into association with the US, such that the CS and context are ‘competing’ for associative strength (Miller & Matzel, 1988; Rescorla & Wagner, 1972; Stout & Miller, 2007; Wagner, 1981). As contextual stimuli generally represent poor predictors of the US, compared to the agent’s actions, the salience of contextual stimuli would typically decline (Le Pelley, 2004; Le Pelley et al., 2016; Mackintosh, 1975; Pearce & Hall, 1980; Pearce & Mackintosh, 2010). However, if redundant stimuli retain greater salience in high schizotypy individuals and people with schizophrenia, the salience of contextual stimuli should remain higher for a longer duration. As elevated salience increases the associability of stimuli and the US is partially contingent upon the context, the association between the context and US would ostensibly be relatively stronger in high schizotypy individuals and people with schizophrenia, which may consequently limit the associative strength acquired by the agent’s actions (Le Pelley, 2004; Le Pelley et al., 2016; Mackintosh, 1975; Miller & Matzel, 1988; Pearce & Hall, 1980; Pearce & Mackintosh, 2010; Rescorla & Wagner, 1972; Stout & Miller, 2007; Wagner, 1981). Attenuating the associative strength of the agent’s actions would theoretically reduce the individual’s perceived ability to modulate environmental contingencies and, by definition, their PC, culminating in an increased risk of depression. Accordingly, the theory that people with schizophrenia assign aberrant salience to redundant stimuli can be extended to contextual stimuli to theoretically account for the manifestation of depression in schizophrenia.

Given that this represents a novel extension of the Salience Hypothesis, it is unsurprising that few studies have evaluated the relationship between DS and the attribution of salience to stimuli. To the best of the author’s knowledge, only one study has examined the relationship between LI and DS, which reported that LI is intact in people with ‘mild depression’ (Msetfi, Byrom & Murphy, 2017). However, participants classed as ‘mildly depressed’ entailed a minimum Beck’s Depression Inventory score of 5, which falls within the range of scores indicative of ‘minimal depression’ (Beck, Steer & Brown, 1996). Thus, there may have been too little variation in DS to detect meaningful differences in LI. Furthermore, participants received pre-exposure to the context in the non-pre-exposed group, which may have resulted in the context acquiring inhibitory properties, by virtue of its presentation in the absence of the US, and/or the CS’s salience being bolstered at test through novel pop-out effects. This would enhance associative learning in the non-pre-exposed group, artificially inflating the magnitude of ‘LI.’ Whilst this study provides limited insight into the relationship between LI and DS, additional research examining the relationship between DS and LI, CI or PL is absent.”

**References**

Anwyl-Irvine, A. L., Massonnié, J., Flitton, A., Kirkham, N., & Evershed, J. K. (2020). Gorilla in our midst: An online behavioral experiment builder. *Behavior Research Methods*, *52*(1), 388–407. https://doi.org/10.3758/s13428-019-01237-x

Ballegooijen, W., Riper, H., Cuijpers, P., Oppen, P., & Smit, J. H. (2016). Validation of online psychometric instruments for common mental health disorders: A systematic review. *BMC Psychiatry*, *16*(1), 1–12. https://doi.org/10.1186/s12888-016-0735-7

Bell, V., Halligan, P. W., & Ellis, H. D. (2006). The Cardiff Anomalous Perceptions Scale (CAPS): A new validated measure of anomalous perceptual experience. *Schizophrenia Bulletin*, *32*(2), 366–377. https://doi.org/10.1093/schbul/sbj014

Burch, G. S. J., Steel, C., & Hemsley, D. R. (1998). Oxford—Liverpool inventory of feelings and experiences: Reliability in an experimental population. *British Journal of Clinical Psychology*, *37*(1), 107–108. https://doi.org/10.1111/j.2044-8260.1998.tb01284.x

Chouinard, V. A., Shinn, A. K., Valeri, L., Chouinard, P. A., Gardner, M. E., Asan, A. E., & Öngür, D. (2019). Visual hallucinations associated with multimodal hallucinations, suicide attempts and morbidity of illness in psychotic disorders. *Schizophrenia Research*, *208*, 196–201. https://doi.org/10.1016/j.schres.2019.02.022

Cochrane, M., Petch, I., & Pickering, A. D. (2010). Do measures of schizotypal personality provide non-clinical analogues of schizophrenic symptomatology? *Psychiatry Research*, *176*(2–3), 150–154. https://doi.org/10.1016/j.psychres.2009.01.031

Cosco, T. D., Prina, M., Stubbs, B., & Wu, Y. T. (2017). Reliability and validity of the Center for Epidemiologic Studies Depression Scale in a population-based cohort of middle-aged US adults. *Journal of Nursing Measurement*, *25*(3), 476–485. https://doi.org/10.1891/1061-3749.25.3.476

Craig, T. J., & Natta, P. A. (1976). Recognition of depressed affect in hospitalized psychiatric patients: Staff and patient perceptions. *Diseases of the Nervous System*, *37*(10), 561–566.

Dijk, S. E., Adriaanse, M. C., Zwaan, L., Bosmans, J. E., Marwijk, H. W., Tulder, M. W., & Terwee, C. B. (2018). Measurement properties of depression questionnaires in patients with diabetes: A systematic review. *Quality of Life Research*, *27*(6), 1415–1430. https://doi.org/10.1007/s11136-018-1782-y

Durbin, J., & Watson, G. S. (1951). Testing for serial correlation in least squares regression II. *Biometrika*, *38*(1/2), 159–178. https://doi.org/10.1093/biomet/38.1-2.159

Field, A. (2013). *Discovering statistics using IBM SPSS statistics* (3rd ed.). Sage.

Friedman, M. (1937). The use of ranks to avoid the assumption of normality implicit in the analysis of variance. *Journal of the American Statistical Association*, *32*(200), 675–701. https://doi.org/10.1080/01621459.1937.10503522

González-Roz, A., Gaalema, D. E., Pericot-Valverde, I., Elliott, R. J., & Ades, P. A. (2019). A systematic review of the diagnostic accuracy of depression questionnaires for cardiac populations: Implications for cardiac rehabilitation. *Journal of cardiopulmonary rehabilitation and prevention*, *39*(6), 354. https://doi.org/10.1097/HCR.0000000000000408

Goulding, A. (2004). Schizotypy models in relation to subjective health and paranormal beliefs and experiences. *Personality and Individual Differences*, *37*(1), 157–167. https://doi.org/10.1016/j.paid.2003.08.008

Jiang, L., Wang, Y., Zhang, Y., Li, R., Wu, H., Li, C., & Tao, Q. (2019). The reliability and validity of the center for epidemiologic studies depression scale (CES-D) for Chinese university students. *Frontiers in Psychiatry*, *10*, 315. https://doi.org/10.3389/fpsyt.2019.00315

Joode, J. W., Dijk, S. E., Walburg, F. S., Bosmans, J. E., Marwijk, H. W., Boer, M. R., & Adriaanse, M. C. (2019). Diagnostic accuracy of depression questionnaires in adult patients with diabetes: A systematic review and meta-analysis. *PLOS One*, *14*(6), 0218512. https://doi.org/10.1371/journal.pone.0218512

Lakens, D. (2013). Calculating and reporting effect sizes to facilitate cumulative science: A practical primer for t-tests and ANOVAs. *Frontiers in Psychology*, *4*. https://www.frontiersin.org/articles/10.3389/fpsyg.2013.00863

Larøi, F., & Linden, M. (2005). Nonclinical Participants’ Reports of Hallucinatory Experiences. *Canadian Journal of Behavioural Science/Revue canadienne des sciences du comportement*, *37*(1), 33–43. https://doi.org/10.1037/h0087243

Lavis, Y., & Mitchell, C. (2006). Effects of preexposure on stimulus discrimination: An investigation of the mechanisms responsible for human perceptual learning. *Quarterly Journal of Experimental Psychology*, *59*(12), 2083–2101. https://doi.org/10.1080/17470210600705198

Lin, A., Wigman, J. T., Nelson, B., Wood, S. J., Vollebergh, W. A., Os, J., & Yung, A. R. (2013). Follow-up factor structure of schizotypy and its clinical associations in a help-seeking sample meeting ultra-high risk for psychosis criteria at baseline. *Comprehensive Psychiatry*, *54*(2), 173–180. https://doi.org/10.1016/j.comppsych.2012.06.011

Lipps, G. E., Lowe, G. A., & Young, R. (2007). Validation of the beck depression inventory-II in a Jamaican university student cohort. *West Indian Medical Journal*, *56*(5), 404–408. https://doi.org/10.1590/s0043-31442006000600011

Mason, O. (1995). A confirmatory factor analysis of the structure of schizotypy. *European Journal of Personality*, *9*(4), 271–281. https://doi.org/10.1002/per.2410090404

Mason, O., & Claridge, G. (2006). The Oxford-Liverpool Inventory of Feelings and Experiences (O-LIFE): Further description and extended norms. *Schizophrenia Research*, *82*(2–3), 203–211. https://doi.org/10.1016/j.schres.2005.12.845

Mason, O., Claridge, G., & Jackson, M. (1995). New scales for the assessment of schizotypy. *Personality and Individual Differences*, *18*(1), 7–13. https://doi.org/10.1016/0191-8869(94)00132-C

Mason, O. J. (2015). The Assessment of Schizotypy and Its Clinical Relevance. *Schizophrenia Bulletin*, *41*(suppl_2), S374–S385. https://doi.org/10.1093/schbul/sbu194

Mauchly, J. W. (1940). Significance test for sphericity of a normal n-variate distribution. *The Annals of Mathematical Statistics*, *11*(2), 204–209. https://doi.org/10.1214/aoms/1177731915

McGrath, J. J., Saha, S., Al-Hamzawi, A., Alonso, J., Bromet, E. J., Bruffaerts, R., & Kessler, R. C. (2015). Psychotic experiences in the general population: A cross-national analysis based on 31 261 respondents from 18 countries. *JAMA Psychiatry*, *72*(7), 697–705. https://doi.org/10.1001/jamapsychiatry.2015.0575

Menard, S. (2002). *Applied logistic regression analysis* (Vol. 106).

Mitchell, C. A., Maybery, M. T., Russell-Smith, S. N., Collerton, D., Gignac, G. E., & Waters, F. (2017). The structure and measurement of unusual sensory experiences in different modalities: The Multi-Modality Unusual Sensory Experiences Questionnaire (MUSEQ. *Frontiers in Psychology*, *8*, 1363. https://doi.org/10.3389/fpsyg.2017.01363

Nagelkerke, N. J. (1991). A note on a general definition of the coefficient of determination. *Biometrika*, *78*(3), 691–692. https://doi.org/10.1093/biomet/78.3.691

Nuevo, R., Chatterji, S., Verdes, E., Naidoo, N., Arango, C., & Ayuso-Mateos, J. L. (2012). The continuum of psychotic symptoms in the general population: A cross-national study. *Schizophrenia Bulletin*, *38*(3), 475–485. https://doi.org/10.1093/schbul/sbq099

Pechey, R., & Halligan, P. (2012). Prevalence and correlates of anomalous experiences in a large non‐clinical sample. *Psychology and Psychotherapy: Theory, Research and Practice*, *85*(2), 150–162. https://doi.org/10.1111/j.2044-8341.2011.02024.x

Radloff, L. S. (1977). The CES-D scale: A self-report depression scale for research in the general population. *Applied Psychological Measurement*, *1*(3), 385–401. https://doi.org/10.1177/014662167700100306

Segal, D. L., Coolidge, F. L., Cahill, B. S., & O’Riley, A. A. (2008). Psychometric properties of the Beck Depression Inventory—II (BDI-II) among community-dwelling older adults. *Behavior Modification*, *32*(1), 3–20. https://doi.org/10.1177/0145445507303833

Skorikov, V. B., & VanderVoort, D. J. (2003). Relationships between the underlying constructs of the Beck Depression Inventory and the Center for Epidemiological Studies Depression Scale. *Educational and Psychological Measurement*, *63*(2), 319–335. https://doi.org/10.1177/0013164402251035

Startup, M. (1999). Schizotypy, dissociative experiences and childhood abuse: Relationships among self‐report measures. *British Journal of Clinical Psychology*, *38*(4), 333–344. https://doi.org/10.1348/014466599162908

Steegen, S., Tuerlinckx, F., Gelman, A., & Vanpaemel, W. (2016). Increasing transparency through a multiverse analysis. *Perspectives on Psychological Science*, *11*(5), 702–712. https://doi.org/10.1177/1745691616658637

Vilagut, G., Forero, C. G., Barbaglia, G., & Alonso, J. (2016). Screening for depression in the general population with the Center for Epidemiologic Studies Depression (CES-D): A systematic review with meta-analysis. *PLOS One*, *11*(5). https://doi.org/10.1371/journal.pone.0155431

Vollema, M. G., & Bosch, R. J. (1995). The multidimensionality of schizotypy. *Schizophrenia Bulletin*, *21*(1), 19–31. https://doi.org/10.1093/schbul/21.1.19

Wang, T., & Mitchell, C. J. (2011). Attention and relative novelty in human perceptual learning. *Journal of Experimental Psychology: Animal Behavior Processes*, *37*(4), 436–445. https://doi.org/10.1037/a0023104

Wang, Y. P., & Gorenstein, C. (2013). Assessment of depression in medical patients: A systematic review of the utility of the Beck Depression Inventory-II. *Clinics*, *68*(9), 1274–1287. https://doi.org/10.6061/clinics/2013(09)15

Wilcoxon, F. (1945). Some uses of statistics in plant pathology. *Biometrics Bulletin*, *1*(4), 41–45. https://doi.org/10.2307/3002011

Yang, L., Jia, C. X., & Qin, P. (2015). Reliability and validity of the Center for Epidemiologic Studies Depression Scale (CES-D) among suicide attempters and comparison residents in rural China. *BMC Psychiatry*, *15*(1), 1–8. https://doi.org/10.1186/s12888-015-0458-1
